# Oral and Vaginal Epithelial Cell Lines Bind and Transfer Cell-Free Infectious HIV-1 to Permissive Cells but Are Not Productively Infected

**DOI:** 10.1371/journal.pone.0098077

**Published:** 2014-05-23

**Authors:** Arinder Kohli, Ayesha Islam, David L. Moyes, Celia Murciano, Chengguo Shen, Stephen J. Challacombe, Julian R. Naglik

**Affiliations:** 1 Department of Oral Immunology, Clinical and Diagnostic Sciences, King's College London Dental Institute, King's College London, London, United Kingdom; 2 Department of Obstetrics and Gynecology, Boston University School of Medicine, Boston, Massachusetts, United States of America; 3 Department of Microbiology and Ecology, University of Valencia, Valencia, Spain; The Hospital for Sick Children and The University of Toronto, Canada

## Abstract

The majority of HIV-1 infections worldwide are acquired via mucosal surfaces. However, unlike the vaginal mucosa, the issue of whether the oral mucosa can act as a portal of entry for HIV-1 infection remains controversial. To address potential differences with regard to the fate of HIV-1 after exposure to oral and vaginal epithelium, we utilized two epithelial cell lines representative of buccal (TR146) and pharyngeal (FaDu) sites of the oral cavity and compared them with a cell line derived from vaginal epithelium (A431) in order to determine (i) HIV-1 receptor gene and protein expression, (ii) whether HIV-1 genome integration into epithelial cells occurs, (iii) whether productive viral infection ensues, and (iv) whether infectious virus can be transferred to permissive cells. Using flow cytometry to measure captured virus by HIV-1 gp120 protein detection and western blot to detect HIV-1 p24 gag protein, we demonstrate that buccal, pharyngeal and vaginal epithelial cells capture CXCR4- and CCR5-utilising virus, probably via non-canonical receptors. Both oral and vaginal epithelial cells are able to transfer infectious virus to permissive cells either directly through cell-cell attachment or via transcytosis of HIV-1 across epithelial cells. However, HIV-1 integration, as measured by real-time PCR and presence of early gene mRNA transcripts and *de novo* protein production were not detected in either epithelial cell type. Importantly, both oral and vaginal epithelial cells were able to support integration and productive infection if HIV-1 entered via the endocytic pathway driven by VSV-G. Our data demonstrate that under normal conditions productive HIV-1 infection of epithelial cells leading to progeny virion production is unlikely, but that epithelial cells can act as mediators of systemic viral dissemination through attachment and transfer of HIV-1 to permissive cells.

## Introduction

The majority of HIV-1 infections worldwide are acquired via mucosal surfaces, predominantly across the female or male genital tracts [1]. Heterosexual transmission accounts for the majority of new HIV-1 infections, and both men and women have been shown to have detectable HIV-1 in seminal fluid and cervicovaginal secretions [2]–[4]. Studies have shown that cell-free [5]and cell-associated [6] HIV-1 can establish mucosal infection and macaque and human studies indicate that transmission is facilitated by the presence of HIV-1 target cells (dendritic cells, Langerhans cells, CD4^+^ T cells and macrophages) in the ectocervix and vagina as well as in the endocervix and uterus [7]–[21]. In contrast, HIV-1 transmission through the oral mucosa is thought to be uncommon [22]–[27]. We and others have shown that several mechanisms may account for the lack of HIV-1 transmission across the oral mucosa, including neutralizing antibodies in seropositive individuals and innate anti-HIV inhibitory factors in saliva and/or epithelium [28]–[32]. However, studies in primates indicate that oral transmission can occur since non-traumatic oral exposure to SIV results in regional dissemination followed by systemic infection [33]–[36]. Therefore, although the oral epithelium may present a barrier to HIV-1 transmission via direct infection, it may also be a conduit for viral entry. This is particularly important given the occurrence of viral transmission in nursing infants and during oro-genital contact in adults.

Entry of HIV-1 into permissive host cells requires expression of the receptor CD4 and a fusion co-receptor (chemokine receptors CCR5 (R5-tropic) or CXCR4 (X4-tropic)) [37]. However, the vast majority of reports indicate that epithelial cells do not express CD4 [38]–[42] and express CCR5 and CXCR4 at either undetectable or very low levels [38], [41], [43]–[47], although data for CXCR4 surface expression is somewhat varied [45], [48]. Despite these receptor dependencies, HIV-1 may also infect CD4^−^ cells and may thus utilize several alternative receptor mechanisms for binding and entry into cells. Besides binding to canonical entry receptors, the viral envelope protein gp160 (gp120 and gp41) also binds to several other cell-surface molecules including DC-SIGN (dendritic cell-specific intercellular adhesion molecule-3-grabbing non-integrin) [49], [50], GalCer (glycosphingolipid galactosylceramide) [51]–[53], and heparan sulphate proteoglycans (HSPGs) such as syndecan-1 [54], [55]. GalCer and HSPGs are commonly expressed on epithelial cells and may promote HIV-1 binding and transport across the oral and vaginal epithelium [32], [46]–[48], [55]. Importantly, there is a preference for R5-tropic viral transmission across mucosal surfaces [56], but a full and satisfactory explanation for this has not yet been provided.

One mechanism of HIV-1 transmission across the mucosa is thought to occur through sequestration of the virus by epithelial cells, followed by transfer to permissive cells to establish a primary infection [7]–[10], [12]–[14], [16]–[18], [47], [57]. Similarly, HIV-1 binding to epithelial cells may directly impair barrier integrity, thus facilitating entry [58], [59]. Indeed, the outermost epithelial layers of the ectocervix and vagina lack tight junctions and are permeable to high molecular weight immunological mediators [60] and, therefore, possibly to virions. However, the fundamental issue of whether epithelial cells can be productively infected with HIV-1 remains controversial. Whilst some studies support the view that HIV-1 can integrate into the vaginal epithelial genome and produce progeny virus [5], [45], [61]–[64], others discount this view [18], [20], [47], [55]. Likewise, in the oral cavity, proviral DNA has been detected in oral epithelial cells [65] and the presence of HIV-1 gag RNA has been demonstrated in both buccal cells [66], [67] and oral biopsies [67], [68]. Furthermore, using primary gingival epithelial cells, one study showed susceptibility to R5 but not X4 tropic viral strains in a CD4-independent manner [69], whilst another study showed X4 rather than R5 susceptibility [38], with epithelial cells being able to secrete infectious virus. Primary epithelial cells isolated from adenoids, palatine tonsils and salivary glands may also be productively infected with HIV-1 [69]–[74]. However, others have found no evidence of productive HIV-1 infection in oral epithelial cells [40], [41]. Rather, HIV-1 is thought to be preferentially sequestered in cytosolic endocytic compartments or transferred to permissive cells after transcytosis across epithelial cells or traverse the epithelial barrier dissimulated in infected cells to establish a primary infection [1], [41], [46], [47], [55], [75]–[80]. Thus, the literature remains ambiguous as to whether HIV-1 proviral DNA integrates into the epithelial cell genome and whether productive infection ensues. Notably, a mechanism has been described recently to explain the difference between oral transmission in adults and infants using *ex vivo* cell models, whereby adult epithelium coats HIV-1 particles with human β-defensins 2 and 3, rendering the cell-free virus less infectious [81]. The same group also established that the paucistratified fetal and infant oral epithelium was more permissive to HIV-1 transcytosis as compared with healthy multistratified adult oral epithelium [82].

Given the ambiguous literature on this fundamentally important topic, we aimed to compare oral and vaginal epithelial cells using identical assay systems to determine whether cell-free HIV-1 binds, enters and integrates into epithelial cells, whether productive infection ensues, and whether sequestered infectious virus can be transferred to permissive cells. We utilized epithelial cell lines representative of two oral sites that are rarely investigated with regard to oral transmission but are one of the first cell types that are likely to come into contact with HIV-1 in the oral cavity and compared them with female genital epithelium. Buccal (TR146), pharyngeal (FaDu) and vulvo-vaginal (A431) epithelial cells, all from stratified squamous cell origin, were utilized to permit convenient and direct comparisons to be made with regard to HIV-1 life cycle events. We demonstrate that oral and vaginal epithelial cells were able to capture X4 and R5 virus but that integration of the viral genome into epithelial cell DNA and *de novo* virus production were not detected. However, VSV-G-packaged HIV-1 was replication competent in all epithelial cell lines. Notably, the three epithelial cell types were able to transfer infectious virus to permissive cells either directly or after transcytosis of HIV-1 across the epithelium, which *in vivo* would permit infection of immune cells in the sub-mucosa and dissemination of HIV-1 in the body.

## Methods

### Ethics Statement

Primary epithelial cells (gingival) were obtained from wisdom tooth extractions and were kindly provided by Maxine Partridge and collection approved by the Guy's Research Ethics Committee. Informed written consent was obtained from each participant.

### Cell lines, primary cells, viruses and virus-like particles

Human oral buccal (TR146) and pharyngeal (FaDu) and vulvovaginal (A431) carcinoma cell lines and 293T (renal epithelial) cells were obtained from the American Type Culture Collection and European Collection of Cell Cultures (ECACC). A431 epithelial cells have different reported origins (vaginal epithelial or epidermal) but are routinely used to represent the vaginal mucosa [83]–[87]. Human glioma cells (NP2) expressing human CD4 and CXCR4 or CCR5 have been previously described [88]. The following reagents were obtained through the AIDS Research and Reference Reagent Program, Division of AIDS, NIAID, NIH: TZM-bl cells (catalogue no. 8129), PM-1 cells (catalogue no. 3038), C8166 T cells (catalogue no. 404), JTLRG-R5 (catalogue no. 11586) and HIV-1 molecular clones pYU2 (R5-utilizing, catalogue no. 1350) and pLAI.2 (X4-utilizing, catalogue no. 2532). Peripheral blood mononuclear cells (PBMCs) were isolated from apheresis cones (National Blood Service Tooting, London, UK) by centrifugation over a Ficoll-Paque density gradient (GE-Healthcare UK Ltd, Little Chalfont, UK). The HIV-1 *gag-pol* expression vector p8.91 and the vesicular stomatitis virus envelope protein (VSV-G) expression vector pMDG were kindly provided by Didier Trono (University of Geneva, Switzerland). The HIV gp160 envelope vectors (pHXB2 (X4), pYU2 (R5) and pSVIII 89.6 (dual tropic)) were a gift from Professor Greg Towers, University College London. The retroviral packaging vector pCSGW encoding green fluorescent protein (GFP) was a kind gift from Adrian Thrasher, Institute of Child Health, University College London, UK. All cell lines were maintained in Dulbecco's Modified Eagle's Medium (DMEM, PAA) supplemented with 10% fetal bovine serum (FBS) (PAA), 100 U of penicillin per mL, and 100 µg of streptomycin per mL (PAA) at 37°C and 5% CO_2_. NP2 cells were additionally supplemented with 1 mg/mL of G418 (Invitrogen) and 1 µg/mL of puromycin (Invitrogen).

### Virus preparation

Viral vectors used for production of VSV-G and HIV gp160 pseudotyped HIV were prepared by transient transfection of 293T cells using a protocol adapted from Besnier *et al*. [89]. Briefly, 293T cells were seeded at 95% confluency in a 10 cm^2^ dish and the following day cells were transfected with 3 µg each of pMDG and p8.91 and 4.5 µg of pCSGW using the polyanionic transfection reagent Jet PEI (Polyplus Transfection) according to the manufacturer's instructions. After 24 h the media was replaced and 48, 72 and 96 h post-transfection virion-containing culture supernatants were harvested and filtrated through a 0.45 µm pore size membrane and stored in aliquots at –80°C until required. Production of infectious stocks of live virus was performed by transient transfection of 293T cells as described above, with 5 µg of the infectious molecular clone pLAI.2 (X4) and or pYU2 (R5) used per transfection. For trypsin sensitivity experiments and detection of integration into epithelium, YU2 virus was grown in NP2-R5 and JLTRG-R5 cells while LAI virus was grown in C8166 cells for 1- 2 weeks with addition of fresh medium until cells showed cytopathic effects and were then harvested and frozen in aliquots.

### Virus titration

Infectious virus stock (LAI and YU2) titers were determined by plaque assay. Briefly, TZM-bl cells (1×10^4^ cells/well) were cultured overnight (96-well plates) and incubated with eight replicates of ten serial dilutions (0.5 log) of virus stock in a total of 100 µL growth media per well. After 48 h, virus supernatant was removed and the cells were fixed with 0.05% glutaraldehyde for 5 min at room temperature and washed twice with phosphate-buffered saline (PBS). Expression of β-galactosidase was determined by staining cells with X-Gal stain [1 mg/mL X-Gal in 5 mM KFe_4_(CN_6_) 3H_2_O, 5 mM KFe_3_(CN_6_) 3H_2_O, and 1 mM MgCl_2_] and incubating culture plates at 37°C for 2 h. Virus infectivity was estimated as plaque forming units (PFU) per mL. Titration of VSV-G pseudotyped HIV-1 and HIV gp160 pseudotyped HIV-1 was carried out using 293T cells or NP2 cells, respectively. Cells were seeded at 1 x 10^5^ cells/well (24-well plates) and cultured overnight at 37°C. Serial dilutions (1∶2) of virus supernatant were applied to the cells (500 µL) and incubated overnight. The following day the media was exchanged and 48 h after transduction with virion-containing culture supernatants the percentage of GFP-expressing cells was determined by flow cytometry using the FACSCanto machine (BD Biosciences). Data was analyzed with FACSDiva software and WinMDI (copyright 1993–2000 Joseph Trotter http://facs.scripps.edu) to calculate the infectious units per mL.

### HIV-1 receptor expression by quantitative reverse transcription-PCR

RNA was isolated from resting TZM-bl, TR146, FaDu and A431 cells using GenElute Mammalian Total RNA Miniprep Kit (Sigma), followed by treatment with Turbo DNA free DNAse (Ambion) according to the manufacturer's instructions. All samples were confirmed DNA free prior to analysis. cDNA was synthesized from 1 µg of RNA using HIV reverse transcriptase (Ambion) according to the manufacturer's instructions. Primers were obtained from RTPrimerDB (http://medgen.ugent.be/rtprimerdb/) and PrimerBank (http://pga.mgh.harvard.edu/primerbank) [90], [91]. Gene expression of CD4, CCR5, CXCR4, DC-SIGN, SDC-1 (syndecan-1) and SDC-4 (syndecan-4) was quantified by real-time PCR using SYBR Green JumpStart Taq Ready Mix (Sigma) with 4 pmol primers and 1 µL cDNA in 10 µL reactions on the Corbett Research Rotor-Gene 6000 (Qiagen) using the following cycling parameters: 95°C for 3 min; followed by 95°C for 3 s, annealing for 10 s and extension for 20–30 s for 40 cycles. Data was analyzed with Corbett Research Rotor-Gene 6000 Series Software 1.7 using the two standard curve method with β-actin used as the normalizer gene. Primer sequences, annealing and extension temperatures are listed in Table 1.

**Table 1:** Primer sets detecting gene expression of HIV-1 associated receptors.

Gene	Primer	Sequence	Annealing temp. (°C)	Extension temp. (°C)	Product (bp)
CD4	Forward Reverse	5′- ACTAAAGGTCCATCCAAGCTGA —3′ 5′- GCAGTCAATCCGAACACTAGCA —3′	60	75	151
CCR5	Forward Reverse	5′- TGGACCAAGCTATGCAGGTG —3′ 5′- CGTGTCACAAGCCCACAGAT —3′	58	75	240
CXCR4	Forward Reverse	5′- CCTCATCCTGGCTTTCTTCG —3′ 5′- GAATGTCCACCTCGCTTTCC —3′	60	75	285
DC-SIGN	Forward Reverse	5′- TCAAGCAGTATTGGAACAGAGGA —3′ 5′- CAGGAGGCTGCGGACTTTTT —3′	60	75	136
Syndecan-1	Forward Reverse	5′- TGAAACCTCGGGGGAGAATAC —3′ 5′- GGTACAGCATGAAACCCACC —3′	60	75	171
Syndecan-4	Forward Reverse	5′- CAGGGTCTGGGAGCCAAGT —3′ 5′- GCACAGTGCTGGACATTGACA —3′	58	72	129
β-actin	Forward Reverse	5′- CATGTACGTTGCTATCCAGGC —3′ 5′- CTCCTTAATGTCACGCACGAT —3′	58	75	250

### HIV-1 receptor expression by flow cytometry

TZM-bl, NP2-X4, NP2-R5, TR146, FaDu and A431 resting cells were washed with PBS and incubated with 0.02% (W/V) EDTA for 5–30 min. Detached cells were washed thoroughly with PBS supplemented with 1% BSA and 0.01% azide (wash buffer), and resuspended at 1×10^6^ cells in 1 mL wash buffer. To identify surface expressed HIV-1 receptors and co-receptors, 100 µL of cells were incubated at room temperature for 1 h with mouse anti-human CD4 (1∶4, catalogue no. 724), CCR5 (1∶20, catalogue no. 4090), CXCR4 (1∶80, catalogue no. 4083), DC-SIGN (1∶100 catalogue no. 6884) monoclonal antibodies (all obtained from the AIDS Research and Reference Reagent Program), GalCer (1∶200, anti-galactocerebroside, Millipore) or heparan sulfate proteoglycan (1∶200, Millipore) monoclonal antibodies. Primary antibodies were detected with goat anti-mouse IgG conjugated with fluorescein isothiocyanate (FITC) (Jackson ImmunoResearch). After thorough washing, cells were fixed in 200 µL 4% formaldehyde and the percentage of FITC-expressing cells was determined by flow cytometry.

### Detection of HIV-1 binding and replication by Western Blot

TR146, FaDu, A431 and TZM-bl cells were seeded at 5 x 10^5^ cells per well and the following day incubated with YU2 (R5) or LAI (X4) virus at a multiplicity of infection (MOI) of 0.2. After overnight incubation at 37°C the cells were washed to remove unbound virus. Cells were harvested in 250 µL 1x RIPA buffer [50 mM Tris/HCl (pH 7.4), 150 mM NaCl, 1% Triton X-100, 1% sodium deoxycholate and 0.1% SDS, supplemented with Halt complete protease inhibitor cocktail (Perbio Science)], placed on ice for 30 min, and stored at −80°C until required. Total protein lysates (mammalian and viral) were normalized for protein content using the bicinchoninic acid (BCA) assay (Pierce) and separated using 12% SDS-PAGE gels. Proteins were transferred to PVDF membranes, probed with anti-HIV-1 gag monoclonal antibody recognizing p24 and p55 isoforms (catalogue no. 6457)) and secondary goat anti-mouse IgG horseradish peroxidase (HRP)-conjugated antibody (Jackson ImmunoResearch), before developing using Immobilon-ECL (Millipore). α-actin was used as a loading control.

### Detection of HIV-1 binding and packaged viral RNA by PCR

TR146, FaDu, A431 and TZM-bl cells were seeded at 5 x 10^5^ cells per well and the following day exposed to YU2 (R5) or LAI (X4) virus at an MOI of 0.2. After overnight incubation at 37°C the cells were washed to remove unbound virus. Total RNA was isolated as above and confirmed DNA free prior to analysis. Equal amounts of total RNA were used to detect packaged viral RNA by first synthesizing cDNA using Superscript cDNA Synthesis Kit (Invitrogen) and an HIV-1 specific primer (5'-GTC ATG AAA CAA ACT TGG C-3'). A 2 µL aliquot of cDNA was then subjected to nested PCR using primers to amplify a 2 kb fragment of the HIV *pol* gene. First round PCR was performed in a 20 µL reaction containing 1x PCR buffer, 100 µM dNTP's, 1.5 mM MgSO_4_, 2.5 U *Taq* polymerase (New England Biolabs) and 10 pmol of each primer (Forward: 5'-AAT GAT GAC AGC ATG TCA GGG AGT-3'; Reverse: 5'-AGT CTT TCC CCA TAT TAC TAT GCT TTC-3'). Cycle parameters were as follows: 95°C for 5 min; 94°C for 10 s, 55°C for 30 s, and 72°C for 1 min for 30 cycles; and an extension of 72°C for 10 min. For subsequent nested PCR, 1 µL of the first round PCR reaction was used as a template to amplify an internal region of the *pol* gene and was performed in a 10 µL reaction containing 1x SYBR Green JumpStart Taq Ready Mix (Sigma), and 3 pmol of each primer (Forward: 5'-TTC TTC AGA GCA GAC CAG-3'; Reverse: 5'-ACT TTT GGG CCA TCC ATT-3'). Cycle parameters were 95°C for 3 min; followed by 95°C for 1 min, 55°C for 30 s, and 72°C for 1 min for 35 cycles; and an extension of 72°C for 10 min. PCR products were resolved on a 2% agarose gel and visualized by ethidium bromide staining.

### Whole virus binding and trypsin sensitivity

TZM-bl, TR146, FaDu and A431 cells (5 x 10^4^) were incubated with either YU2 (R5) or LAI (X4) virus at an MOI of 5 overnight at 4°C. Cells were washed three times with PBS and blocked in PBS/10% BSA for 10 min at room temperature. To determine whether HIV-1 binding was trypsin sensitive, prior to blocking, cells were treated with trypsin (0.05%)-EDTA (0.02%) (PAA, UK) for 5 min at 37°C. Cells were gently removed by scraping and labeled with HIV-1 gp120 monoclonal antibodies F425 A1g8 and F425 B4e8 (1∶200) (AIDS Research and Reference Reagent Program) followed by Cy5-conjugated AffinityPure goat anti-human IgG secondary antibody (1∶400) (Jackson ImmunoResearch), each for 30 min at 4°C. Cells were washed three times with PBS, resuspended in 4% formaldehyde and subjected to flow cytometry. Binding percentages for whole virus were calculated as increased Cy5 shift of HIV-1 infected labelled cells from uninfected labelled cells. Given the higher background with a new batch of F425 B4e8 antibody, binding percentages for the trypsin sensitivity data were calculated as increased Cy5 shift relative to HIV-1 infected secondary alone labelled cells.

### Detection of HIV-1 integration by primer-probe Alu-LTR PCR assay

To determine whether HIV DNA was able to integrate into epithelial cells a real-time PCR assay was performed with HIV-1 LTR and human Alu-specific primers with a U5 specific probe as previously described [92]. TR146, FaDu, A431 and PM-1 (control) cells were seeded at 5 x 10^5^ cells per well and the following day exposed to YU2 (R5) or LAI (X4) virus, pre-treated with RNAse-free DNAse (Roche, UK) at 37°C for 1 h with 4 mM MgCl_2_. MOI's ranged from 1 to 140. Heat-inactivated virus (60°C for 1 h) without DNAse treatment was used as a DNA contamination control. Cells and virus were incubated for 48 h at 37°C, after which cells were washed three times with PBS before DNA was extracted using the GenElute Mammalian Genomic DNA Miniprep Kit (Sigma, Poole, UK) according to the manufacturer's instructions with Proteinase K digestion for 20 min. Isolated DNA samples were digested with DpnI (New England Biolabs, UK) to degrade any plasmid DNA contaminant. DNA was then quantified by Nanodrop and either 50 ng or 100 ng DNA was analysed by real-time PCR on a Rotorgene 6000 (Qiagen, UK) using primers 0.2 M MH535 forward (5′-AACTAGGGAACCCACTGCTTAAG-3′) and 0.8 M reverse SB704 (5′-TGCTGGGATTACAGGCGTGAG-3′) with 0.2 M probe P-HUS-SS1 (5′FAM-TAGTGTGTGCCCGTCTGTTGTGTGAC-TAMRA-3′) using Jumpstart Ready Mix (Sigma, Poole, UK) in 10 µL reactions. For each PCR reaction the same concentration of DNA was used for all samples isolated from individual cell lines. Samples were denatured for 10 min followed by 60 cycles of 94°C for 15 s, 60°C for 30 s and 72°C for 60 s. DNAse-treated virus exposed samples were compared with heat-inactivated virus exposed samples. Positive integration events were taken as a lower C_t_ in the DNAse treated virus exposed sample than the heat-inactivated virus control.

### Productive viral infection by detection of spliced HIV-1 *tat* by PCR

TR146, FaDu, A431 and TZM-bl cells were seeded at 5 x 10^5^ cells per well and the following day incubated with YU2 (R5) or LAI (X4) virus at an MOI of 0.2. After overnight incubation at 37°C unbound virus was removed by washing and total RNA isolated (GenElute Mammalian Total RNA Miniprep Kit; Sigma). Genomic DNA was removed with Turbo DNAse free (Ambion) according to the manufacturer's instructions and samples were confirmed DNA free prior to analysis. Equal amounts of total RNA was used to synthesize viral cDNA transcripts using the HIV-specific oligo ART-7 5′- TTC TAT TCC TTC GGG CCT GTC G -3′. A 1 µL aliquot of cDNA was then subjected to PCR using primers spanning the *tat*1 and 2 exon junctions (*tat*-junction forward: 5′- TAG ATC CTA GAC TAG AGC CC-3′ and *tat*-junction reverse 5′- TTG GGA GGT GGG TCT GAA ACG-3′) in a 20 µL reaction containing 1x PCR buffer, 100 µM dNTP's, 1.5 mM MgSO_4_, 2.5 U *Taq* polymerase (New England Biolabs) and 10 pmol of each primer. Cycle parameters were as follows: 95°C for 5 min; followed by 94°C for 10 s, 55°C for 30 s, and 72°C for 1 min for 35 cycles; with a final extension of 72°C for 10 min. PCR products were resolved on a 2% agarose gel and visualized by ethidium bromide staining.

### HIV-1 integration and productive infection using pseudotyped virus-like particles

TR146, FaDu, A431, NP2-R5 and NP2-X4 cells were seeded at 1 x 10^5^ cells per well and cultured overnight at 37°C. Serial dilutions (1∶2) of HIV-1 gp160 pseudotyped (X4, R5 and dual tropic) and VSV-G pseudotyped HIV-1 were applied and incubated overnight at 37°C. HIV-1 integration and *de novo* virus protein production were determined by the presence of GFP-expressing cells by flow cytometry. To inhibit HIV-1 specific GFP expression, infections were also carried out in the presence of 500 µM of the HIV-1 reverse transcriptase inhibitor AZT (NIH AIDS Reagent Program Cat no. 3485).

### Detection of *de novo* HIV-1 production by indicator cell infection

TR146, FaDu, A431 and TZM-bl cells were seeded at 5 x 10^5^ cells per well and the following day cultured with YU2 (R5) or LAI (X4) virus at an MOI of 0.2. After overnight incubation at 37°C the cells were extensively washed with HBSS (Invitrogen) to remove unbound virus. Fresh media was applied to the cells and the plates were incubated at 37°C for up to 7 days to allow any *de novo*-produced infectious virus to be released into the medium. Culture medium (potentially containing infectious virus) was then applied to 3 x 10^5^ TZM-bl indicator cells and incubated for a further 24 h at 37°C. Cells were fixed, washed twice with PBS and stained for β-galactosidase expression with X-Gal stain. Individual wells were visualized by light microscopy at 100 X magnification.

### HIV-1 transfer assay

FaDu, TR146, A431 and TZM-bl cells were seeded at 1 x 10^5^ cells and the following day exposed to YU2 (R5) or LAI (X4) virus at an MOI of 0.2. After overnight incubation at 37°C the cells were thoroughly washed in HBSS to remove any unbound virus. Controls included FaDu and TR146 and A431 cells without the addition of virus. TZM-bl cells (3 x 10^5^) were then overlaid onto the epithelial cells and the plates incubated for a further 48 h at 37°C. Cells were fixed and stained for β-galactosidase expression with X-Gal stain. Individual wells were photographed by light microscopy at 100 X magnification.

### HIV-1 transcytosis across epithelium

TR146, FaDu and A431 cells were seeded at 5 x 10^4^cells per Millipore transwell 24-well inserts (membrane pore size 0.4 µm) and the following day (after confluency was reached) incubated with YU2 (R5) or LAI (X4) virus in phenol red-free medium at an MOI of 0.2 containing 2.3 mg/mL of dextran blue [59], [93]. After 4 h incubation at 37°C, the cells in inserts were thoroughly washed with PBS to remove any unbound virus. Controls included epithelial cells without the addition of virus. The transwell inserts were then overlaid upon a confluent monolayer of TZM-bl cells in 24-well plates (the epithelial cells were separated from the TZM-bl cells by the transwell membrane preventing cell-cell contact). Plates were incubated for a further 48 h at 37°C to allow HIV-1 to transcytose across the epithelial cells and through the membrane pores to infect the underlying TZM-bl cells. TZM-bl cells were fixed, stained for β-galactosidase expression with X-Gal stain, and counted using light microscope at 100 X magnification.

### Statistical analysis

Where shown, the data were analyzed by ANOVA using SigmaPlot 12.5 (Systat Software Inc). A p value of less than 0.05 was taken to be significant.

## Results

### Expression of HIV-1 receptors in epithelial cells

**Figure 1:**
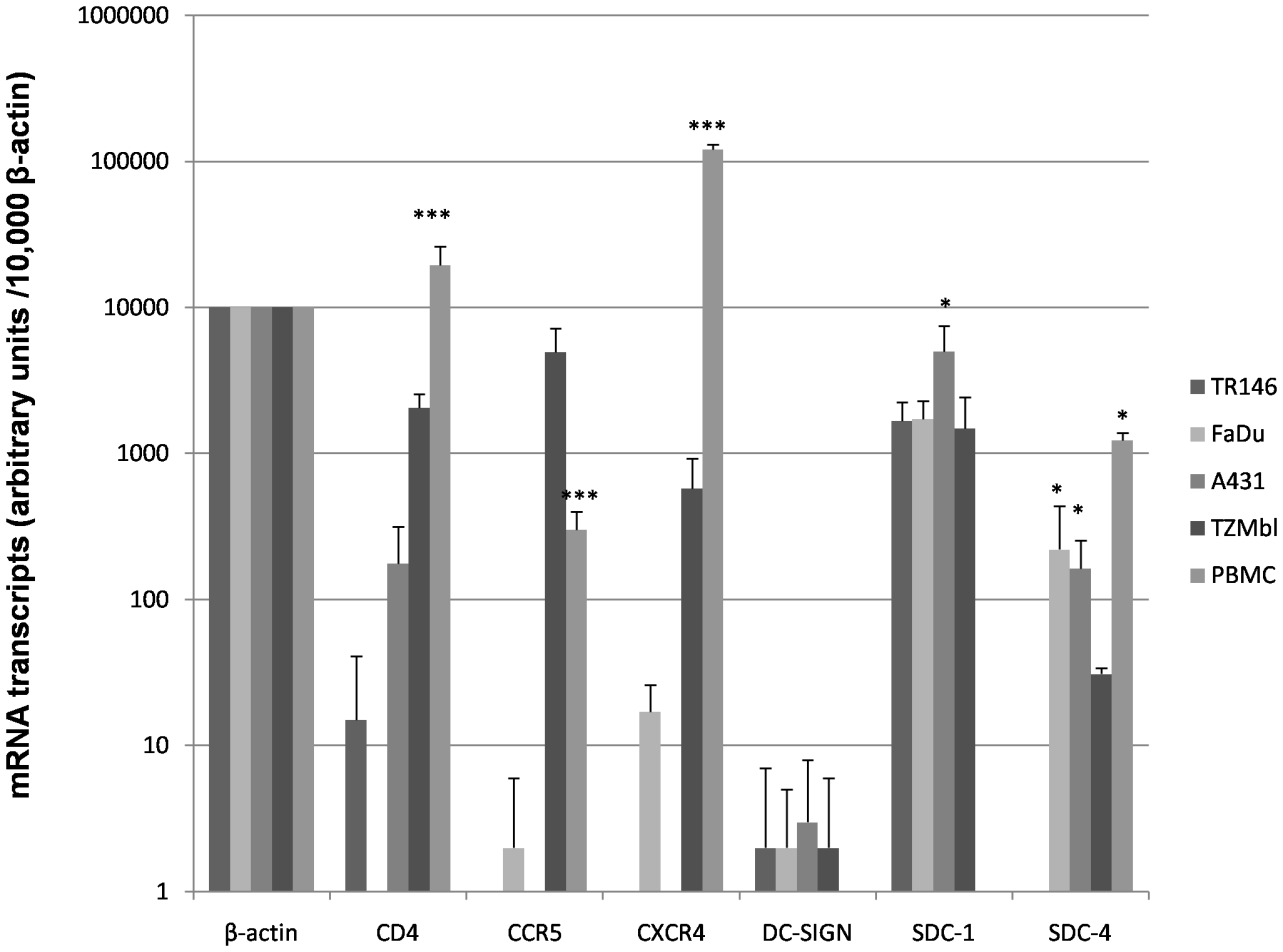
Basal HIV-1 receptor mRNA expression in resting epithelial cells. TR146, FaDu, A431 and TZM-bl cells were examined for mRNA expression of CD4, CCR5, CXCR4, DC-SIGN and the HSPG's syndecan-1 and -4 by quantitative RT-PCR. Data are presented as mRNA transcripts (arbitrary units) normalized to β-actin in a minimum of three independent experiments. PBMCs showed significantly higher expression of CD4, CCR5 and CXCR4 than oral (TR146 and FaDu) or vaginal (A431) cell lines. A431 cells show significantly higher expression of SDC-1, whilst FaDu and A431 show significantly higher expression of SDC-4 than TR146. Bars indicate ± standard deviation from the mean. *** = *P*<0.001, * = *P*<0.05.

We first analyzed the gene expression levels of canonical (CD4, CCR5, CXCR4) and non-canonical (DC-SIGN and HSPG's, excluding GalCer as this non-protein moiety cannot be investigated by gene expression) HIV-1 receptors in epithelial cells by quantitative PCR (Figure 1). This demonstrated the absence or minimal expression of CD4, CCR5 and CXCR4 in both TR146 (buccal) and FaDu (pharyngeal) cells compared with PBMCs, (*P*<0.001). Similar data were obtained with the A431 (vaginal) cells but notably CD4 mRNA was detected, albeit at levels approximately 110-fold lower than PBMCs (*P*<0.001). Control TZM-bl cells exhibited greater (*P*<0.001) expression of all three genes relative to oral and vaginal cell lines but still lower expression of CD4 (10-fold lower) and CXCR4 (200-fold lower) than PBMCs (*P*<0.001). Expression of CCR5 in TZM-bl cells was greater than 10-fold higher than PBMCs (*P*<0.001). In all three cell types DC-SIGN was minimally expressed but HSPG syndecan-1 was highly expressed, particularly in A431 cells (*P*<0.05). The only major difference observed between the epithelial cell types was the expression of the HSPG syndecan-4 in FaDu and A431 cells, which was undetectable in TR146 cells (*P*<0.05).

**Figure 2:**
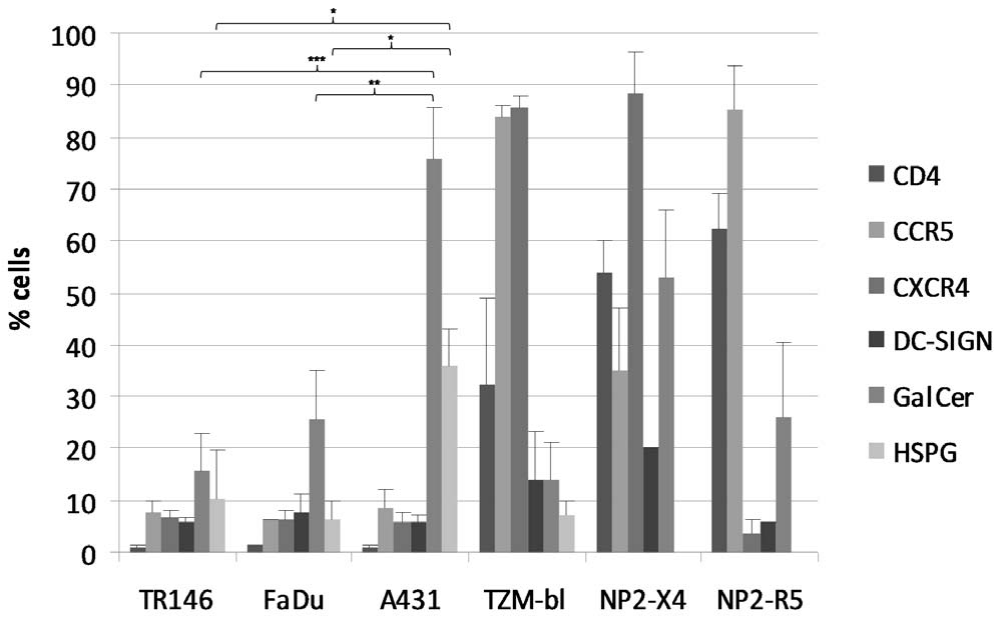
Basal HIV-1 receptor surface expression in resting epithelial cells. TR146, FaDu, A431, TZM-bl and NP2-R5 and –X4 expressing cells were examined for surface expression of CD4, CCR5, CXCR4, DC-SIGN, GalCer and HSPG's by flow cytometry using monoclonal primary antibodies specific to each receptor with a FITC-labeled secondary antibody. HSPG's were not analyzed in NP2-R5 or -X4 expressing cells. Data are presented as percentage of cells expressing each receptor in a minimum of three independent experiments. Bars indicate ± standard error of the mean. *** = *P*<0.001, ** = *P*<0.01, * = *P*<0.05.

We next determined the surface expression (% positive cells) of CD4, CCR5, CXCR4, DC-SIGN, GalCer, and HSPG's on epithelial cells by flow cytometry (Figure 2 and Figure S1). All three cell lines expressed undetectable levels of CD4 and very low levels of CCR5 and CXCR4 (∼6–8%) compared with control TZM-bl cells, which are HeLa cell derivatives engineered to express CD4 (32%), CCR5 (82%) and CXCR4 (85%). NP2 cells expressing either CCR5 (85%) or CXCR4 (88%) were also used as positive controls and expressed high levels of CD4 (50–60%). With regard to non-canonical receptors, both TR146 and FaDu oral cells expressed similar amounts of DC-SIGN (6–8%) and HSPG's (6–10%) but FaDu cells expressed greater amounts of GalCer than TR146 cells (25% and 15%, respectively). The similar amounts of surface HSPG's expressed on TR146 and FaDu cells appears to be in contrast to the differences in gene expression data for syndecan-4 (Figure 1); however, this was expected since the detecting antibody recognizes other HSPG's including syndecan-1, which is highly expressed in both TR146 and FaDu cells (Figure 1). Notably, A431 vaginal cells expressed significantly greater surface levels of GalCer (∼75%) and HSPG's (35%) compared with the oral cells (TR146 GalCer 15.6% (*P*<0.001), HSPG ∼10.3% (*P*<0.05); FaDu GalCer 25.5% (*P*<0.01), HSPG 6.3% (*P*<0.05)) (Figure 2 and Figure S1). TZM-bl cells expressed low levels of DC-SIGN (14%), GalCer (14%) and HSPG's (7%). Likewise NP2-X4 and –R5 cells expressed low levels of DC-SIGN (5–20%), but higher levels of GalCer (25–50%).

### HIV-1 binding to epithelial cells

We next determined whether HIV-1 can be captured by oral and vaginal epithelial cells. TR146, FaDu, A431 and TZM-bl cells were incubated overnight with cell free YU2 (R5) or LAI (X4) infectious virus. After extensive washing, the presence of attached virus was determined using three separate approaches.

**Figure 3:**
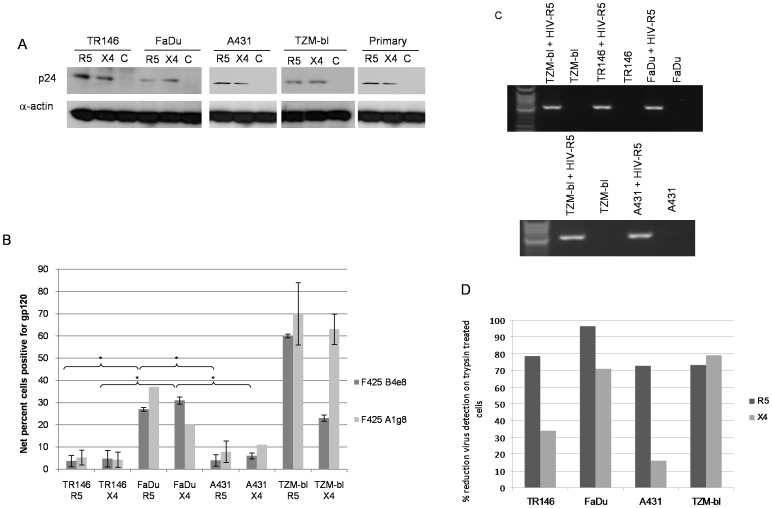
Different methods used to detect HIV-1 R5 and X4 binding to epithelial cells. (A) Post-lysis detection of p24 gag protein by Western blotting. Primary (gingival) epithelial cells, TR146, FaDu, A431 and TZM-bl cells were incubated overnight (16–24 h) with cell free YU2 (R5) or LAI (X4). After extensive washing to remove unbound virus, normalised total protein lysates were separated by SDS-PAGE and probed for HIV p24 using α-actin as a loading control. (B) Detection of immobilized virus on the cell surface by flow cytometry. Epithelial cells were incubated overnight with cell free virus. Bound virus was detected using a Cy5-labeled anti-human secondary antibody to detect HIV-1 gp120 primary monoclonal on the APC channel. Electronic gates were set around an unlabelled cell control, this area is then set as zero and any cells shifted to the right of the gate are deemed positive. To determine amount of virus bound, virally exposed, labelled cell percentages are subtracted from the uninfected (unexposed) labelled control cell percentages to obtain the % fluorescence values shown. Data are representative of four independent experiments and bars indicate ± standard deviation from the mean. (C) Detection of packaged HIV R5 RNA by amplification of the HIV-1 *pol* gene using nested PCR. Total RNA was extracted from TR146, FaDu, A431 and TZM-bl cells incubated overnight with cell free YU2 (R5) and used to produce viral cDNA. This was then used as a template in a nested PCR to detect a 2 Kb region of HIV pol. (D) Percentage reduction in detection of immobilized virus on the cell surface by flow cytometry after trypsin treatment. Virally exposed cells are compared with cells labelled with secondary antibody alone. Data set is representative of three independent experiments. * = *P*<0.05.

First, total protein was isolated and the presence of HIV-1 p24 gag protein determined by immunoblot analysis. p24 was present in TR146, FaDu and A431 protein lysates at levels similar to that found with TZM-bl cells, indicating that both R5 and X4 virus are captured by both oral and vaginal epithelial cells (Figure 3A). We confirmed that R5 and X4 virus are also captured by primary oral epithelial cells (gingival) (Figure 3A). Given the identical HIV-1 binding data between primary and carcinoma epithelial cells, all other experiments were performed with TR146, FaDu and A431 cells.

Second, using a more quantitative approach, the presence of immobilized virus on the surface of TR146, FaDu and A431 cells was determined by flow cytometry using a Cy5-labeled secondary antibody to detect a human monoclonal primary (F425 B4e8) that detected HIV-1 gp120. Both R5 and X4 virus was detected on TR146, FaDu and A431cells demonstrating direct binding of infectious virus to both oral and vaginal epithelial cells (Figure 3B). However, FaDu cells bind significantly more of both R5 (27%) and X4 (31%) virus than TR146 (R5 4%, (*P*<0.05), X4 5% (*P*<0.05)) and A431 (R5 4.5% (P<0.05), X4 5.5%, (*P*<0.05)) cells but to a lesser degree than control TZM-bl cells (62–68%). A second monoclonal primary antibody (F425 A1g8) showed similar binding to the epithelial lines as F425 B4e8 (Figure 3B and Figure S2).

Third, we also determined the presence of captured virus through the detection of packaged HIV RNA by amplification of the HIV-1 *pol* gene using nested PCR. This approach was used to confirm the p24 protein and whole virus binding data; therefore, we performed this experiment using R5 virus only. Amplification of the HIV-1 *pol* gene indicated the presence of R5 virus on TR146, FaDu and A431 cells in addition to TZM-bl cells (Figure 3C). The Western blot (Figure 3A) and PCR (Figure 3C) data are qualitative and may not reflect differences in the efficiency of R5 and X4 HIV-1 binding to TR146, FaDu or A431 cells, which was more apparent when using the quantitative flow cytometry approach (Figure 3B).

Finally, we determined whether binding (performed at 4°C) of both R5 and X4 virus to TR146, FaDu and A431 cells was trypsin sensitive. Notably, R5 binding to all three cell lines was highly sensitive to trypsin with a reduction of between 73–97% binding, whereas X4 virus appeared more trypsin resistant, especially in TR146 and A431 cells where viral binding was reduced only by 33% and 16%, respectively (Figure 3D). This suggests that R5 virus interacts predominantly with trypsin-sensitive protein moieties on epithelial cells to mediate binding, whilst X4 virus also utilizes trypsin-insensitive or additional non-protein moieties. Taken together, the data demonstrate that both R5 and X4 virus bind directly to buccal, pharyngeal and vaginal epithelial cells, although differences may exist in the surface moieties used for attachment.

### HIV-1 mRNA transcription and *de novo* viral protein production

Given that HIV-1 was able to bind to TR146, FaDu and A431 cells, we hypothesized that epithelial cells may support productive viral infection. To investigate this we utilized four different approaches.

**Figure 4:**
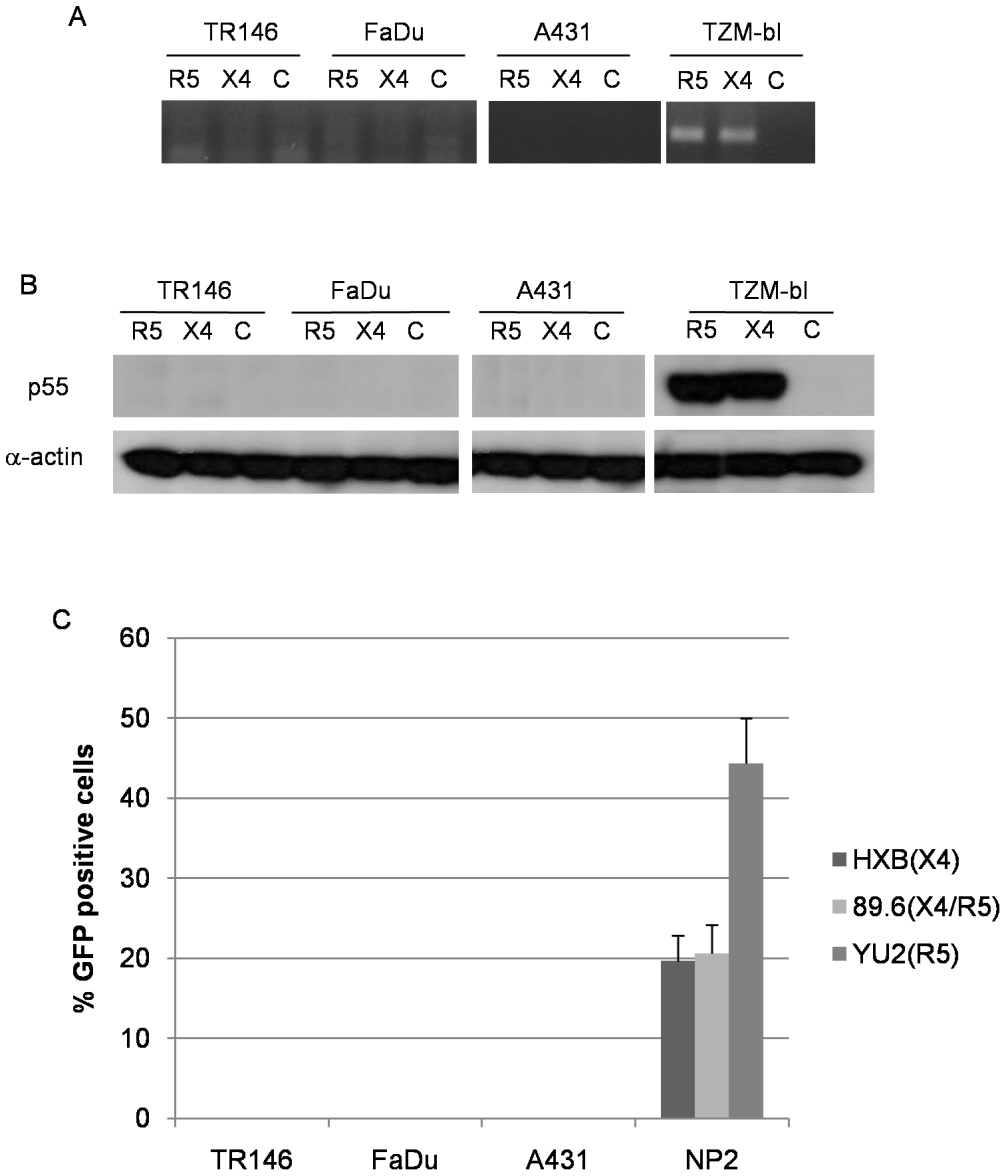
Post-integration HIV-1 mRNA transcription and *de novo* viral protein production in epithelial cells (MOI: 0.2). (A) Detection of spliced HIV-1 *tat* mRNA in TR146, FaDu, A431 and TZM-bl control cells by PCR 24 h post-infection with YU2 (R5) or LAI (X4) infectious virus. Equal amounts of total RNA was used to synthesise viral cDNA which was then subjected to PCR using primers designed to span the TAT 1 and 2 exon junctions. (B) p55 gag protein detection in TR146, FaDu, A431 and TZM-bl control cells by Western blot after 24 h infection with R5 (YU2) and LAI (X4) virus. (C) Infection of TR146, FaDu, A431 and NP2-R5/X4 control cells with GFP-linked single-cycle X4, R5 and dual tropic HIV-1 gp160 pseudotyped virus and detection of GFP incorporation into epithelial cell DNA by flow cytometry. Error bars show standard error from the mean. Data are representative of three independent experiments.

First, we used a PCR-based system to detect spliced HIV-1 *tat* mRNA in the target epithelial cells 24 h post-infection with infectious R5 and X4 virus. The presence of high levels of spliced *tat* mRNA indicates HIV-1 integration and *de novo* production of viral mRNA transcripts, which in permissive cells is representative of a productive HIV-1 infection. However, we found that spliced *tat* mRNA was only detected in control TZM-bl cells but not in TR146, FaDu or A431 cells (Figure 4A).

Second, we assessed protein lysates of TR146, FaDu, A431 and TZM-bl cells for p55 gag protein by Western blot 24 h post-infection with R5 or X4 virus. Detection of p55 gag protein would only be observed if *de novo* viral protein production had occurred. Figure 4B shows p55 gag protein expression only in the control TZM-bl cells but not in the TR146, FaDu or A431 epithelial cells.

Third, since most assays were undertaken 24–48 h post-infection with HIV-1, we also assessed for HIV-1 progeny after day 4 and 7 in case an extended time period was required to establish a productive infection in oral and vaginal epithelial cells. Therefore, after addition of infectious R5 and X4 virus to TR146, FaDu and A431 for 24 h, the cells were thoroughly washed and the medium replenished for up to 7 days. Culture supernatants taken at day 4 and 7 (potentially containing new viral progeny) and were then transferred onto TZM-bl indicator cells for 48 h. Any *de novo* virus production and subsequent infection of TZM-bl cells would cause HIV-LTR driven β-galactosidase production, which would be visible as blue foci in the assay. However, no blue foci were observed. Furthermore, p24 protein in culture supernatants was also absent at day 7 (data not shown). Together the data indicate that no new virions were produced after prolonged virus incubation in oral or vaginal epithelial cells.

Fourth, we utilized the three-plasmid expression system developed by Naldini *et al* [94] to generate HIV-based vectors pseudotyped with HIV-1 envelopes, HXB (X4), YU2 (R5) or 89.6 (dual tropic). Supernatants containing replication defective retroviral particles were used to transduce TR146, FaDu, A431 and NP2-R5/X4 cells. NP2 cells were used as the positive control because the receptor expression is maintained using selective media and thus efficiency of viral infection was greater than with TZM-bl cells. Unlike the HIV canonical receptor expressing NP2 cells, TR146, FaDu and A431 cells failed to drive expression of the GFP reporter gene resulting in undetectable GFP fluorescence up to 48 h post-infection (Figure 4C). Taken together, our extensive data sets indicate that productive HIV-1 infection does not occur in oral or vaginal epithelial cells.

### HIV-1 integration into epithelial cells

Although both R5 and X4 virus was unable to productively infect TR146, FaDu or A431 cells, it was possible that, post-capture, HIV-1 was able to gain entry and integrated into the epithelial cell DNA to establish a latent infection. To test for this possibility we performed a real-time PCR assay to detect integrated viral DNA using primer sets specific for HIV-1 LTR and human Alu sequences with a FAM-TAMRA probe specific for the U5 region of the LTR [92]. Initial experiments performed at an MOI of 1 indicated no integration by R5 or X4 virus in any epithelial cell line after 48 h (data not shown). Further experiments demonstrated that increasing the MOI to 7.5 (X4) and 10 (R5) also failed to permit integration into TR146, FaDu or A431 cells, whereas in the control cell lines (NP2-R5 and C8166) amplification of the integration product was detected after 29 and 32 cycles, respectively (Table 2). To confirm that lack of viral integration into the epithelial cell genome was not due to the presence of insufficient amounts of HIV-1, a final experiment using X4 virus at an MOI of 140 was performed, which also failed to produce detectable levels of HIV-1 integration (Table 2). These data demonstrate that HIV-1 X4 and R5 do not integrate into the oral or vaginal epithelial genome.

**Table 2:** Detection of integrated HIV-1 genome in epithelial cells by qPCR.

Cell line	R5-YU2 (MOI = 7.5)	X4-LAI (MOI = 10)	X4-LAI (MOI = 140)
FaDu	ND	Not included	ND
TR146	ND	ND	ND
A431	ND	ND	ND
C8166^a^	n/a	+ (Ct = 33)	+ (Ct = 33)
NP2-R5^b^	+ (Ct = 29)	n/a	n/a

a C8166 cells express CXCR4 and were used for X4 viral infections only.

b NP2-R5 cells express CCR5 and were used for R5 viral infections only.

+, Integrated HIV-1 product detected (cycle threshold detection in brackets).

ND, Integrated HIV-1 product not detected.

Real-time PCR assay to detect integrated HIV-DNA using HIV-1 LTR and human Alu-specific primers and a U5 specific probe [92]. TR146, FaDu, A431 and PM-1 (control) cells were exposed to YU2 (R5) or LAI (X4) virus for 48 h at 37°C. DNA was extracted, digested with DpnI to degrade any plasmid DNA contaminant and analysed by real-time PCR.

### Productive HIV-1 infection is not restricted when HIV-1 enters via the endocytic pathway

**Figure 5:**
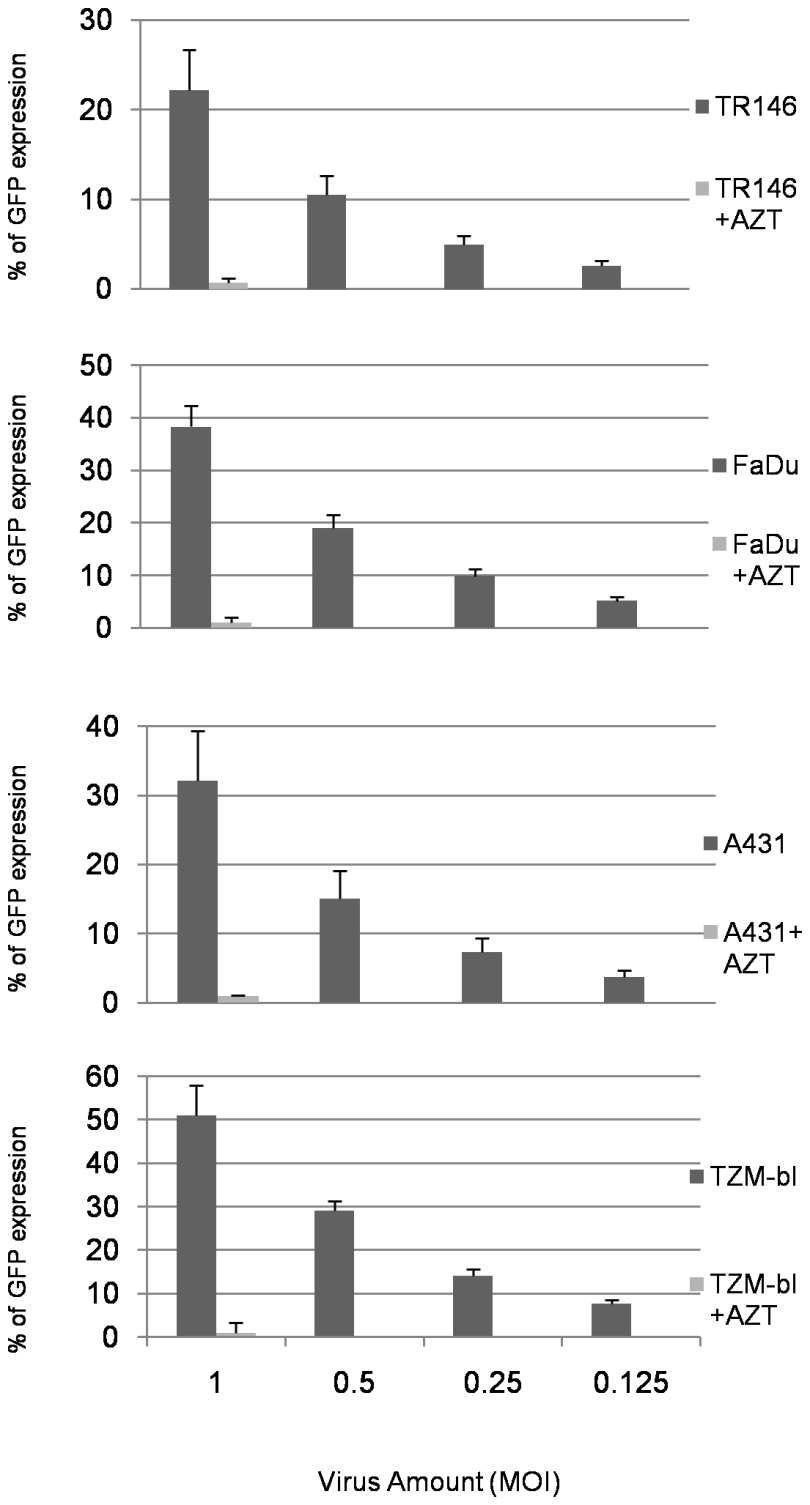
HIV-1 entry via the endocytic pathway results in productive viral infection in epithelial cells. Two fold serial dilutions of VSV-G pseudotyped HIV-1 (MOI 1 –0.125) were added to TR146, FaDu, A431 and TZM-bl control cells. Infection is measured 16–24 h later by flow cytometry as percentage of GFP expression. The effect of AZT (500 mM) on GFP expression was also measured at the highest virus inoculum. Error bars show standard error from the mean. Data are representative of three independent experiments

Although R5 and X4 virus does not integrate and establish a productive infection in TR146, FaDu and A431 cells, we sought to determine whether epithelial cells possessed the cellular machinery to support productive infection if conventional receptor-mediated entry mechanisms were by-passed. Therefore, we utilized the same three-plasmid expression system as described above [94] to generate VSV-G protein-pseudotyped HIV-1 vectors encoding GFP. By utilizing the endocytic entry of VSV-G, strong GFP fluorescence was observed by flow cytometry in TR146 (22%), FaDu (38%) and A431 cells (32%) epithelial cells, at levels only moderately lower than TZM-bl cells (50%) (Figure 5). The differences in infection efficiency between the cell lines may be due to the different growth rates of TR146, FaDu and A431 cells. Importantly, the data indicate that epithelial cells are able to support productive HIV-1 infection if HIV-1 enters via the endocytic pathway. Addition of the HIV-1 reverse transcriptase inhibitor AZT abolished GFP fluorescence with the VSV-G pseudotyped virus, indicating the specificity of HIV-1 production in both epithelial cell types.

### HIV-1 transfer from epithelial cells to permissive cells

**Figure 6:**
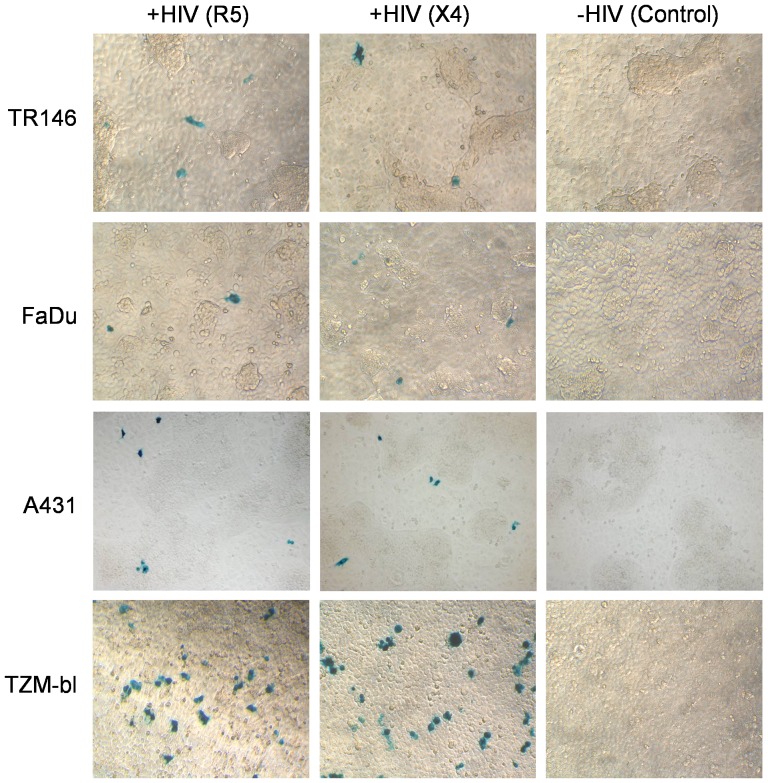
Transfer of captured HIV-1 from epithelial cells to permissive cells via cell-cell contact. TR146, FaDu and A431 cells were incubated with R5 (YU2) and LAI (X4) virus for 24 h and following extensive washing TZM-bl indicator cells were added for a further 48 h. Controls included FaDu, TR146 and A431 cells without the addition of virus. Data are representative of three independent experiments.

One proposed mechanism of HIV-1 transmission across mucosal surfaces is via transfer of infections virus to underlying permissive cells post-capture by epithelial cells. Given that both R5 and X4 virus can be captured by oral and vaginal epithelial cells (Figure 3) but does not result in integration (Table 2) or productive viral infection (Figure 4), we next determined whether immobilized HIV-1 remained infectious post-capture. TR146, FaDu and A431 cells were incubated with R5 and X4 virus for 24 h to allow for viral binding and following extensive washing, TZM-bl indicator cells were added for up to a further 48 h. Transfer of virus from TR146, FaDu and A431 cells to TZM-bl cells and their subsequent infection would result in β-galactosidase activation and the appearance of blue foci. Figure 6 indicates the presence of blue foci, which demonstrates that R5 and X4 virus can be transferred from both oral and vaginal epithelial cells to permissive cells. Experiments were performed with additional controls including absence of HIV-1 and incubation of TZM-bl cells with conditioned medium, none of which resulted in the appearance of blue foci, indicating that the blue foci were the result of R5 and X4 transfer from the epithelial cells.

### HIV-1 transcytosis across epithelium

**Figure 7:**
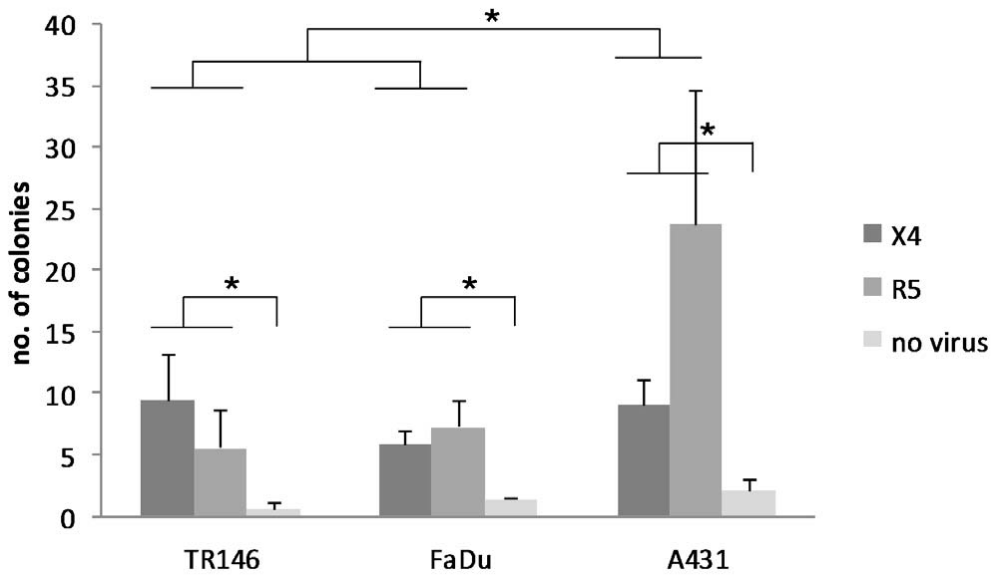
Transfer of captured HIV-1 from epithelial cells to permissive cells via transcytosis. TR146, FaDu and A431 were grown on polycarbonate transwell membranes and incubated with R5 (YU2) and LAI (X4) virus for 4 h. Following extensive washing the transwells were placed in a separate plate that overlaid a confluent monolayer of TZM-bl cells and incubated for a further 48 h at 37°C. TZM-bl cells were fixed, stained for β-galactosidase expression with X-Gal stain, and counted using light microscope at 100x magnification. Resulting replicate colony counts were averaged and analyzed by two factor (cell line and virus tropism) ANOVA and post hoc Fisher PLSD tests. Colony counts through A431 monolayers were significantly greater than those through TR146 and FaDu (*P*<0.05). Colony counts resulting from exposure of epithelium to X4 and R5 were significantly greater (*P*<0.05) than control wells with no virus exposure, but were not significantly different from each other. Data are representative of three independent experiments. * *P*<0.05.

Another proposed mechanism of HIV-1 transmission is via transcytosis across epithelial barriers to infect underlying permissive cells. To test this, we developed a transwell system, similar to the method developed by Nazli *et al* [59], in which R5 and X4 virus that had bound to epithelial cells was separated from permissive TZM-bl cells by an inert membrane. HIV-1 was only capable of infecting the TZM-bl cells if the virus transcytosed across the epithelial cells and through the membrane pores (0.4 µm). Dextran blue was added to all cultures to ensure that the epithelial monolayer remained confluent and that virus did not migrate between 'gaps' in the epithelial cells to the underlying TZM-bl compartment (Figure S3). After 48 h incubation at 37°C, HIV-1 was capable of infecting TZM-bl as demonstrated by the presence of blue foci (Figure 7). The colony counts resulting from exposure of epithelium to X4 (p = 0.036) and R5 (p = 0.001) were significantly greater than control wells with no virus exposure, but were not significantly different from each other. Furthermore, transcytosis through A431 epithelial cells was significantly greater than through TR146 (p = 0.038) and FaDu (p = 0.030) epithelial cells. This demonstrates that HIV-1 is capable of transcytosing across epithelial cells to infect underlying permissive cells and that transcytosis is more efficient across the vaginal epithelium.

It should be noted that a very small amount of blue foci were observed in the absence of HIV-1 (Figure 7). The HIV-1 LTR possesses NF-κB and AP-1 transcription factor binding sites (among other sites). Given that cytokines and growth factors are constitutively produced by resting epithelial cells, low level HIV-1 LTR activation can sometimes be observed (‘leakiness’) in the absence of virus. However, in the presence of HIV-1, cellular transcription factors and machinery at the HIV-1 LTR are stabilised by tat allowing for a greater and more consistent increase in signal, as observed.

## Discussion

The majority of HIV-1 infections worldwide are acquired via mucosal surfaces. Transmission is predominantly across the female genital tract [1], with oral transmission across the oral mucosa being more uncommon [26]. However, given that the oral cavity is a prime site for HIV-1 transmission in nursing infants and during oral-genital contact, it is profoundly important to understand the fate of HIV-1 after exposure to both vaginal and oral tissues and to determine whether differences exist at the two sites. One view is that HIV-1 can directly infect epithelial cells with low efficiency, thereby establishing a primary infection. Another view is that HIV-1 is captured by epithelial cells and is subsequently transferred to permissive cells in the sub-mucosa either directly from the surface or after transcytosis across epithelial cells. In this study we compared both mucosal sites using identical assay systems to study the fate of HIV-1 R5 and X4 virus after exposure to buccal (TR146), pharyngeal (FaDu) and vulvovaginal (A431) epithelial cells. A431 epithelial cells have different reported origins (vaginal epithelial or epidermal) but are routinely used to represent the vaginal mucosa [83]–[87]. Our work strongly supports the second view, whereby oral and vaginal epithelial cells are able to capture and subsequently transfer infectious virus to permissive cells but that viral genome integration and other markers for infectious virus production, such as mRNA transcription and viral protein production, do not occur. However, epithelial cells possess the cellular machinery to support HIV-1 replication since the above-mentioned post-entry processes are not restricted if conventional HIV-1 entry is circumvented through VSV-G mediated endocyctic pathways. Therefore, we propose that oral and vaginal epithelial cells may play an important function in HIV-1 dissemination through their ability to bind HIV-1 and transfer viable virus to permissive cells, which *in vivo* would permit infection of immune cells in the sub-mucosa and establishment of a primary HIV-1 infection.

Using qualitative and quantitative analyses we demonstrate that both R5 and X4 virus are able to bind directly to TR146, FaDu and A431 epithelial cells. Binding appears to be independent of canonical HIV-1 receptor expression, since low or undetectable levels of CD4, CCR5 and CXCR4 expression were found in both oral and vaginal epithelial cell types, which is in concordance with most studies investigating epithelial expression of these receptors [27], [39], [40], [45]–[47], [69], [70]. This also supports studies with primary epithelial cells using inhibitors and neutralising antibodies, which indicate that HIV-1 binding is independent of CD4, CXCR4 and CCR5 [46]. Rather, given their higher surface expression, viral binding is probably mediated via non-canonical receptors such as GalCer and HSPG's (e.g. syndecans) but unlikely to be via DC-SIGN, which was expressed at very low levels in all cell types. Although some studies have suggested that HSPGs rather than GalCer are the key moieties involved in HIV-1 binding to vaginal epithelial cells [46], we found no correlation between increased viral binding with GalCer or HSPG expression in either epithelial cell type. However, we did correlate increased viral binding in FaDu cells with trypsin sensitivity, indicating that FaDu cells may utilize additional surface moieties for viral biding than TR146 or A431 cells.

The utilization of both protein (e.g. HSPG) and non-protein (e.g. GalCer) moieties by HIV-1 to bind epithelial cells is supported by the fact that R5 and X4 binding was reduced but not abolished after trypsin digestion. Notably, X4 virus may preferentially utilize non-protein moieties since a greater number of X4 virus remained attached after trypsin digestion as compared with R5 virus. The reason for this X4 trypsin resistant binding is unclear but may reflect the utilisation of different regions of X4 and R5 gp120 to bind various target receptors or, alternatively, X4 virus may somehow preferentially be protected over R5 virus by other epithelial structures on the surface. While most primary HIV-1 infections occur with R5 virus [95] and some studies have found a preference for R5 selection in oral epithelial cells via cell-associated [70] and cell-free systems [69], our extensive binding data indicate no such preference in vaginal, buccal and pharyngeal epithelial cells as both R5 and X4 virus appear to bind equally well.

Whether viral entry and genome integration occurs in epithelial cells is unclear, but this may be reflective of the variety of experimental approaches and procedures used in different studies. One study using immortalized OKF6/TERT-2 oral cells (floor of mouth) showed that HIV-1 may directly integrate into epithelial cells without establishing a productive infection [41]. However, the lack of DNAse treatment of virus stocks prior to infection or DpnI treatment of DNA samples after infection may not have been sufficient to remove potential plasmid contamination. Although nuclear extracts were used and heat-inactivated controls included, this may not have been sufficient to prevent false positives for viral integration. Another study detected proviral HIV-1 DNA in between 29–68% vaginal and cervical clinical samples, indicating viral integration [64]. However, the samples may have included immune cells that were present in mucosa or secretions at the time of collection, especially since bleeding was observed in approximately 50% of patients upon cervical sample collection. A separate study detected proviral HIV-1 DNA in three carcinoma cell lines (HEC1A, endometrium; CaSki, cervix; SiHa, uterus) and primary vaginal epithelial cells [45] and noted a preference for X4 integration, since all these cells expressed high amounts of CXCR4 (∼60%) and SDF-1 (a CXCR4 ligand) blocked integration. Studies with primary gingival [38] and uterine [96] epithelial cells have also demonstrated preferential integration of X4 virus, probably because of high surface expression of CXCR4 and GalCer, which together can be used as alternative receptors to CD4 for viral entry [38]. However, other studies have failed to observe HIV-1 *gag* DNA in primary cervical epithelial cells or the cervical ME-180 cell line [47], or the ectocervix cell line (Ect1/E6E7) despite HIV-1 being captured and subsequently released [55]. The latter study further confirmed a lack of viral integration using a luciferase reporter virus expressing CCR5 gp120 envelope.

Using a sensitive real-time PCR assay that measures the amount of carry-over DNA, as all samples are quantified relative to a matched heat-inactivated control, we provide strong evidence that both R5 and X4 virus are unable to integrate their genomes into the DNA of oral or vaginal epithelial cells. One explanation for this may be the very low surface expression levels of CXCR4 in TR146, FaDu and A431 cells (∼5%). In this regard, the origin of A431 cells (vulval), which is different to those vaginal epithelial cells used in the above studies, may account for the differences in CXCR4 expression and hence X4 viral integration. Another explanation may be that, unlike the above studies, our samples were rigorously treated with DNAse and DpnI digestion to prevent the detection of possible false positive integration events. Interestingly, another study showed that HIV-1 proviral DNA could be detected in differentiated colonic epithelial cell clones but not undifferentiated clones [63]. If applicable to vaginal epithelial cells, this may have implications for HIV-1 transmission *in vivo*, as the virus may more readily integrate into apical (differentiated) rather than basal (undifferentiated) epithelial cells. This may provide an additional explanation for why proviral DNA was not detected in A431 cells as they are an undifferentiated cell line [97].

To further study the fate of the virus after binding to oral and vaginal epithelial cells and to determine whether HIV-1 can productively infect epithelial cells, we employed three different approaches: infection with HIV-1 gp160 pseudotyped virus, detection of spliced HIV-1 *tat* mRNA, and *de novo* production of p55 gag protein. Using these approaches we demonstrate that HIV-1 infection, *de novo* HIV-1 protein production and viral assembly are not supported in either epithelial cell type. These observations, together with the general absence of CD4/CXCR4/CCR5 expression in both oral and vaginal epithelial cells, support the view that productive HIV-1 infection requires canonical receptor expression on the host cell. Our findings are in concordance with the majority of other studies demonstrating a lack of productive HIV-1 infection in epithelial cells despite the presence of HSPGs and GalCer [17], [20], [47], [55], [98]. However, they are in contrast with other studies demonstrating productive viral infection in epithelial cell lineages isolated from tonsilar tissue [71]–[73], adenoids [69], salivary glands [70], [74] primary gingival keratinocytes [38], [69] and vaginal epithelial cells [5], [61]–[63]. Notably, the findings of the above studies indicating infection appear to correlate with greater expression of CXCR4 and/or GalCer than that found in our study. Finally, the *in vivo* relevance of one study demonstrating productive infection of X4 virus but not R5 virus in primary gingival cells [38] was questioned by Quinones-Mateu [40], as it used the artificial compound polybrene to promote HIV-1 viral entry into the epithelial cell (see below).

As noted above we have also demonstrated that trypsin treatment failed to remove all surface-bound HIV-1. This raises an important issue with regard to other co-culture studies [41], [99] that have claimed infection of permissive cells as a result of *de novo* virus production in epithelial cells. In these studies it is possible that new viral progeny may have originated from trypsin-resistant bound HIV-1, which was transferred to the permissive cells from the epithelial cell surface leading to their infection, and not from *de novo* virus production in epithelial cells.

Several studies have reported that HIV-1 may be sequestered in cytosolic endocytic compartments [1], [46], [77], [79], [80], [100], which may result in productive infection. Whilst one study showed that HIV-1 released in vesicles by infected T-cells were taken up by cervical (ME100) carcinoma epithelial cells resulting in productive infection [62], another study showed a lack of productive infection after 18 days despite integrated proviral DNA being present [45]. To address whether HIV-1 entry via endocytosis results in productive infection we utilized a GFP-encoding VSV-G pseudotyped HIV-1 virus, which utilizes the endocytic pathway for cell entry and by-passes conventional CD4 receptor-mediated entry. This virus was able to establish a productive infection in TR146, FaDu and A431 cells that could be inhibited with AZT, demonstrating that HIV-1 binding in epithelial cells is probably mediated through non-canonical receptors and epithelial cells are able to assemble and secrete infectious viral progeny if receptor-mediated entry is by-passed. Together with the fact that HIV-1 infection of TZM-bl cells (epithelial-like cells expressing CD4, CXCR4 and CCR5) also results in the assembly and secretion of infectious viral progeny, our data suggests that oral and vaginal epithelial cells are able to support productive viral infection, but only if HIV-1 gains entry into the cell through non-conventional (endocytic) mechanisms. This may explain why the use of polybrene led to productive HIV-1 infection in primary gingival epithelial cells [38] (see above). Our findings raise the intriguing possibility that if conditions arise *in vivo* that enable receptor-mediated entry to be by-passed, for example during inflammatory responses, productive HIV-1 infection may be supported in epithelial cells [101].

We propose that under 'normal' conditions it is unlikely that HIV-1 binding results in productive viral infection in epithelial cells. However, post-capture, infectious virus may remain immobilized on the surface giving rise to the possibility of transmission to permissive cells in the underlying mucosa either through direct cell-cell transfer or via viral transcytosis across the epithelial cell. A number of studies support this hypothesis [7]–[14], [16]–[18], [47], [57], [102], although others could not demonstrate viral transfer to permissive cells (PBMCs) by cell-cell contact [45]. Utilizing an overlay experiment we show that HIV-1 retains infectivity on the epithelial cell surface and can be readily transferred to TZM-bl cells via direct cell-cell contact to establish a productive infection. We also demonstrate using a transwell system that HIV-1 can transcytose across oral and vaginal epithelial cells to infect TZM-bl cells separated from the epithelium by a permeable membrane. Transfer of virus to permissive cells has also been observed for other oral epithelial cell lineages [38], [41], [55], [78] and appears to occur in a CD4/CCR5/CXCR4 and GalCer independent manner. Although we are uncertain of the surface moieties that enable binding and transfer, others have demonstrated an important role for HSPGs, since heparin or heparin sulfate can inhibit gp120 binding to CD4 cells and heparinase treatment can reduce viral attachment [54], [55], [103], [104]. These findings have implications for HIV-1 infection *in vivo* as this may provide a window of opportunity for infectious immobilized virus to be transferred to susceptible immune cells in the sub-mucosa, thereby establishing an acute infection and disseminating the virus in the body. This is supported by both macaque and human studies, which indicate that viral transmission is facilitated by the presence of HIV-1 target cells (dendritic cells, Langerhans cells, CD4^+^ T cells and macrophages) in the uterus, endocervix, ectocervix and vagina [7]–[21], [96]. Furthermore, systemic viral dissemination can be observed 24 h after atraumatic exposure of the oral mucosa to SIV-1 [36]. In addition, using *ex vivo* tissue explants Tugizov *et al*. [81], [82] recently demonstrated that HIV-1 efficiently transcytoses through adult and fetal polarized oral epithelial cells, although only virions emerging after transcytosis from fetal epithelial cells appeared to be infectious. Our data are in agreement with the latter studies indicating viral transcytosis through oral and vaginal epithelial cells but additionally that HIV-1 remains infectious enabling transfer to permissive cells basally.

We note that our data is based on cell lines and does not mimic the pluristratified, thre-dimensional structure of the buccal mucosa. However, despite these limitations, our data conform to primary cell studies and support the view that HIV-1 is readily captured by epithelial cells but that genome integration and productive viral infection does not occur. However, epithelial cells possess the cellular machinery to support productive HIV-1 infection if the virus enters via the endocytic pathway or if conventional entry mechanisms are by-passed. Once captured, HIV-1 also remains infectious on the surface of epithelial cells, which may facilitate viral transfer to permissive cells (e.g. dendritic cells, Langerhans cells, tissue macrophages) in the sub-mucosa either directly from the epithelial surface or after transcytosis through the epithelium, thereby establishing acute infection.

## Supporting Information

Figure S1 (TIF)

Figure S2 (TIF)

Figure S3 (TIF)
