# Correction: Oral and Vaginal Epithelial Cell Lines Bind and Transfer Cell-Free Infectious HIV-1 to Permissive Cells but Are Not Productively Infected

**DOI:** 10.1371/journal.pone.0229553

**Published:** 2020-02-19

**Authors:** Arinder Kohli, Ayesha Islam, David L. Moyes, Celia Murciano, Chengguo Shen, Stephen J. Challacombe, Julian R. Naglik

*PLOS ONE* apologizes for our delay in posting this Correction and acknowledges that the authors responded to the concerns about this article by providing data and clarifications in 2015.

After publication of this article [1], concerns were raised about the following blots in Figs 3A and 4B:

- In Fig 3A, the p24 panels for A431 and Primary cell lanes appear to be the same. The authors clarified that the correct data are shown in the published figure for Primary cells, and that this panel was duplicated in error as representing A431 p24 data. The A431 p24 panel is corrected in the updated version of Fig 3 provided with this Correction, and the original images underlying the p24 results for Primary cell and cell line experiments are included in S1–S3 Files.
- The α-actin control panels in Figs 3A and 4B appear the same for TR146, FaDu, and TZM-bl cells. The authors explained that the same blot was used in generating the p24, p55, and α-actin data shown in Figs 3A and 4B for TR146, FaDu, A431, and TZM-bl. Hence the same control blots apply to both figures. This is reflected in the updated figure legends provided with this notice.
- In Fig 3A, the α-actin control panels for A431 and Primary cell lanes appear to be the same as lanes 3, 4, and 5 of the TR146/FaDu α-actin panel. In Fig 4B, the α-actin control panel for A431 appears to be identical to lanes 2, 3, and 4 of the α-actin panel for TR146 and FaDu cell lines. The authors clarified that the wrong α-actin data were shown in the published Fig 3A for A431 and Primary cells and in Fig 4B for A431 cells. This is corrected in the updated figures and the original α-actin blot images for the cell lines and Primary cell α-actin panels are provided in S4 and S5 Files.

**Fig 3:**
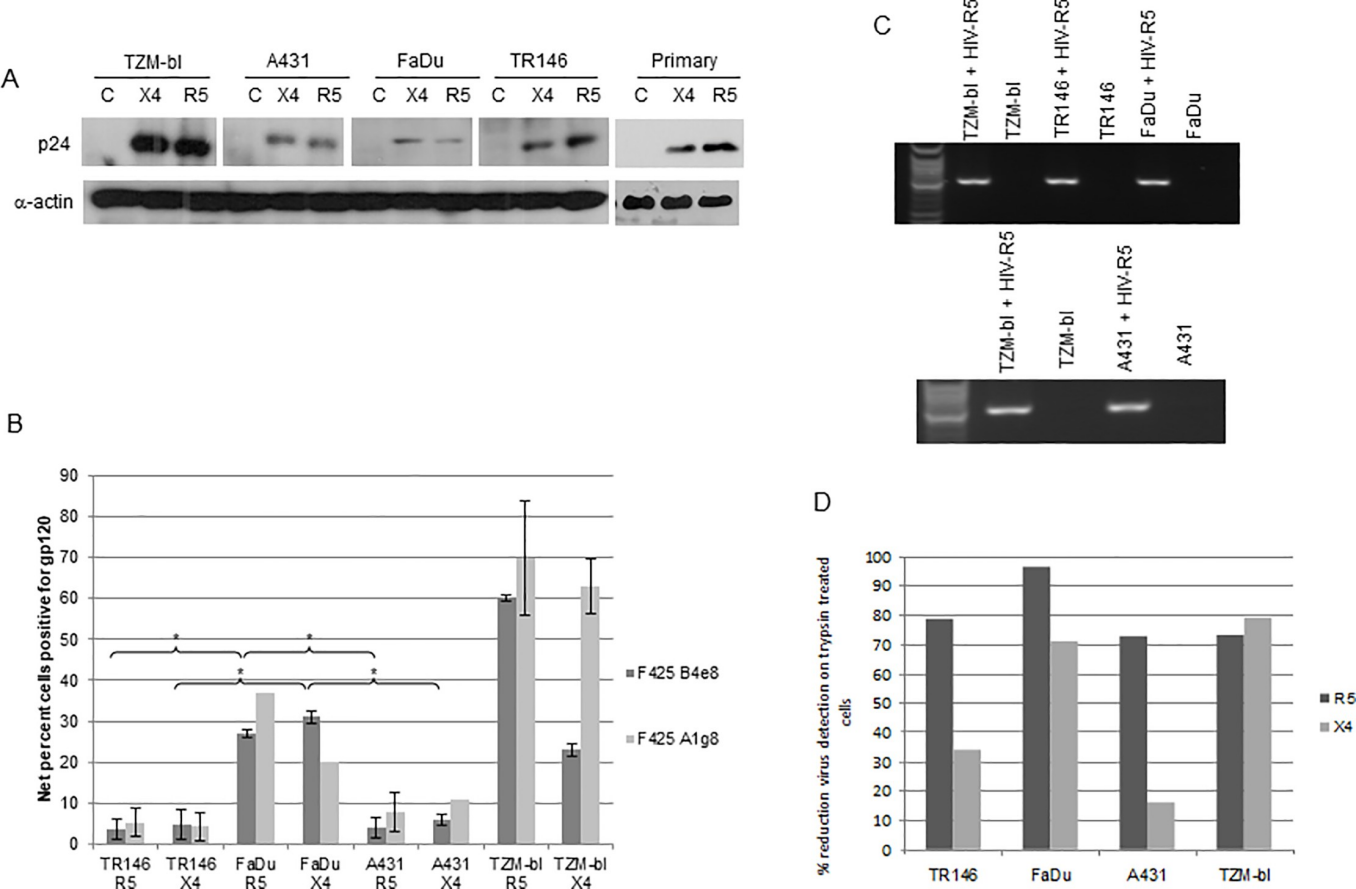
Different methods used to detect HIV-1 R5 and X4 binding to epithelial cells. (A) Post-lysis detection of p24 gag protein by Western blotting. Primary (gingival) epithelial cells, TR146, FaDu, A431 and TZM-bl cells were incubated overnight (16–24 h) with cell free YU2 (R5) or LAI (X4). After extensive washing to remove unbound virus, normalised total protein lysates were separated by SDS-PAGE and probed for HIV p24 using **α**-actin as a loading control. The same membranes were used to obtain cell line data reported in Figs 3A and 4B, and so the same **α**-actin data are shown in the two figures shown. The original image data are matched experimental and controls from the same samples, and are provided in S2–S4 Files. (B) Detection of immobilized virus on the cell surface by flow cytometry. Epithelial cells were incubated overnight with cell free virus. Bound virus was detected using a Cy5-labeled anti-human secondary antibody to detect HIV-1 gp120 primary monoclonal on the APC channel. Electronic gates were set around an unlabelled cell control, this area is then set as zero and any cells shifted to the right of the gate are deemed positive. To determine amount of virus bound, virally exposed, labelled cell percentages are subtracted from the uninfected (unexposed) labelled control cell percentages to obtain the % fluorescence values shown. Data are representative of four independent experiments and bars indicate ± standard deviation from the mean. (C) Detection of packaged HIV R5 RNA by amplification of the HIV-1 *pol* gene using nested PCR. Total RNA was extracted from TR146, FaDu, A431 and TZM-bl cells incubated overnight with cell free YU2 (R5) and used to produce viral cDNA. This was then used as a template in a nested PCR to detect a 2 Kb region of HIV pol. (D) Percentage reduction in detection of immobilized virus on the cell surface by flow cytometry after trypsin treatment. Virally exposed cells are compared with cells labelled with secondary antibody alone. Data set is representative of three independent experiments. *  *= P*<0.05.

**Fig 4:**
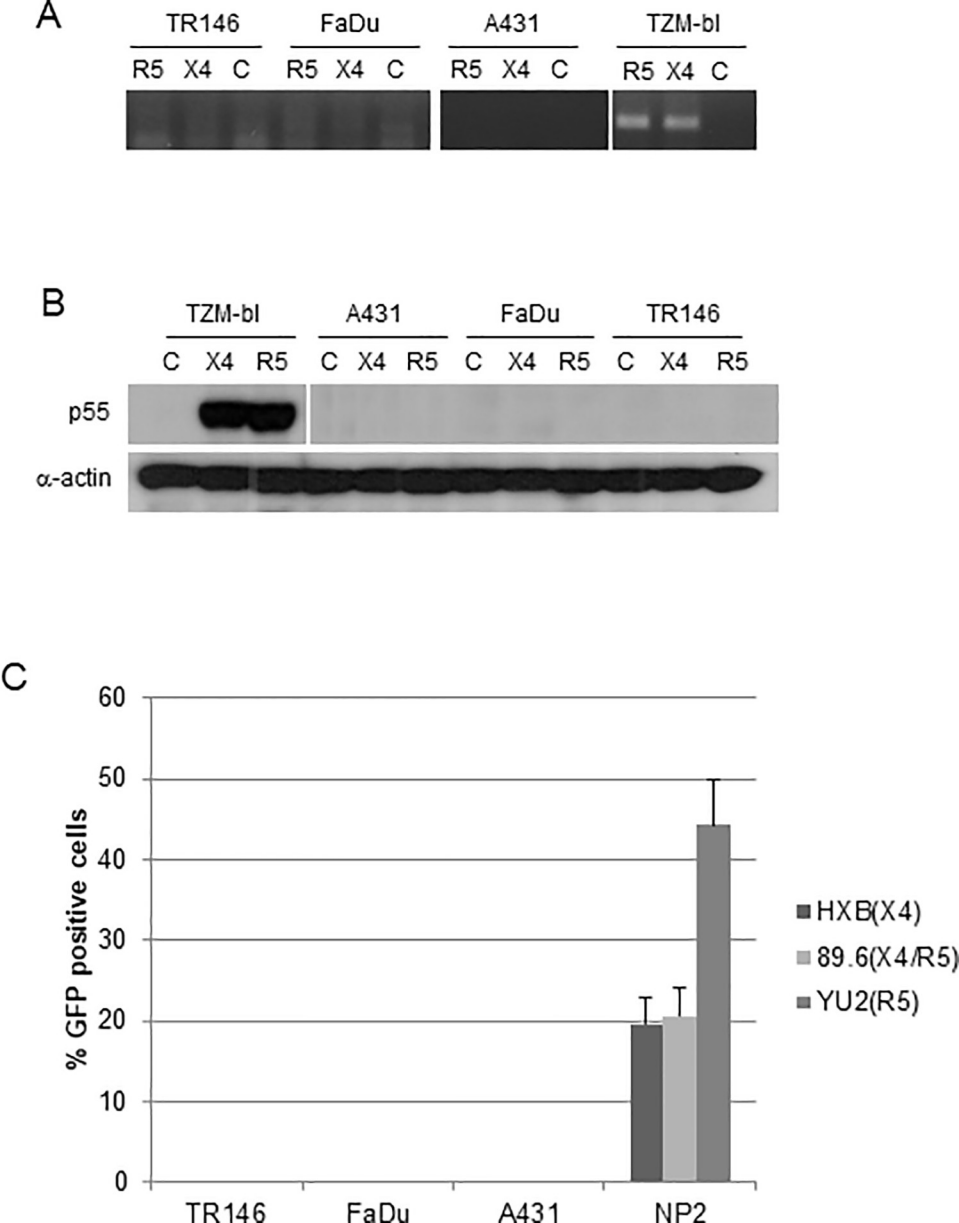
Post-integration HIV-1 mRNA transcription and *de novo* viral protein production in epithelial cells (MOI: 0.2). (A) Detection of spliced HIV-1 *tat* mRNA in TR146, FaDu, A431 and TZM-bl control cells by PCR 24 h post-infection with YU2 (R5) or LAI (X4) infectious virus. Equal amounts of total RNA was used to synthesise viral cDNA which was then subjected to PCR using primers designed to span the TAT 1 and 2 exon junctions. (B) p55 gag protein detection in TR146, FaDu, A431 and TZM-bl control cells by Western blot after 24 h infection with R5 (YU2) and LAI (X4) virus. Data in Fig 4B were obtained using the same samples and blots as those shown in Fig 3A, and therefore the same **α**-actin blot is shown in both figures. The original data are matched experimental and controls from the same samples, original blot images are in S2–S4 Files. (C) Infection of TR146, FaDu, A431 and NP2-R5/X4 control cells with GFP-linked single-cycle X4, R5 and dual tropic HIV-1 gp160 pseudotyped virus and detection of GFP incorporation into epithelial cell DNA by flow cytometry. Error bars show standard error from the mean. Data are representative of three independent experiments.

In reviewing the primary image data, it came to light that the p24 data shown in Fig 3A for TZM-bl cells were obtained using a shorter blot exposure than the data for the other three cell lines (see S2 and S3 Files). The same blot was used to obtain the p24 data for all four cell lines, and the α-actin data for all cell lines reflect the same exposure of the same blot. The TZM-bl panel has been revised in the updated figure so that the p24 data for all four cell lines reflect the same exposure of the same blot (S3 File). The updated figure and supporting raw image data in S2 and S3 Files indicate that p24 was expressed at higher levels in TZM-bl than in the other three cell lines. As a result, statements comparing expression across cell lines are not supported. Specifically, the following sentences (second and fourth) from the second paragraph of the ‘HIV-1 binding to epithelial cells’ subsection of the Results are not supported:

“p24 was present in TR146, FaDu and A431 protein lysates at levels similar to that found with TZM-bl cells, indicating that both R5 and X4 virus are captured by both oral and vaginal epithelial cells (Fig 3A).”

“Given the identical HIV-1 binding data between primary and carcinoma epithelial cells, all other experiments were performed with TR146, FaDu and A431 cells.”

In addition, the Primary cell p24 panel has been updated in the revised Fig 3A so that the aspect ratio in the figure panel aligns more closely with the original blot image (see S1 File).

The authors apologize for the errors in the published article and confirm that the original data underlying other results in this article are available upon request.

## Supporting information

S1 File Original image of the primary cell anti-p24 western blot. The image on the right, with aspect ratio adjusted to compress the image vertically, is shown in the published figure.
(JPG)

S2 File Short exposure of the original blot including p24 and p55 data for cell lines, reported in Figs 3A and 4B, respectively. (JPG)

S3 File Long exposure of the original blot including p24 and p55 data for cell lines, reported in Figs 3A and 4B, respectively. (JPG)

S4 File Original image of the anti-α-actin western blot for cell lines. (JPG)

S5 File Original image of the primary cell anti-α-actin western blot. Data for the primary cell p24 and α-actin experiments were obtained using blots prepared with the same protein preparations.
(JPG)
