# Supplementary figures and images for: Oral and Vaginal Epithelial Cell Lines Bind and Transfer Cell-Free Infectious HIV-1 to Permissive Cells but Are Not Productively Infected
Source: PLoS One. 2014 May 23;9(5):e98077. doi: 10.1371/journal.pone.0098077 (PMC4032250; doi:10.1371/journal.pone.0098077)

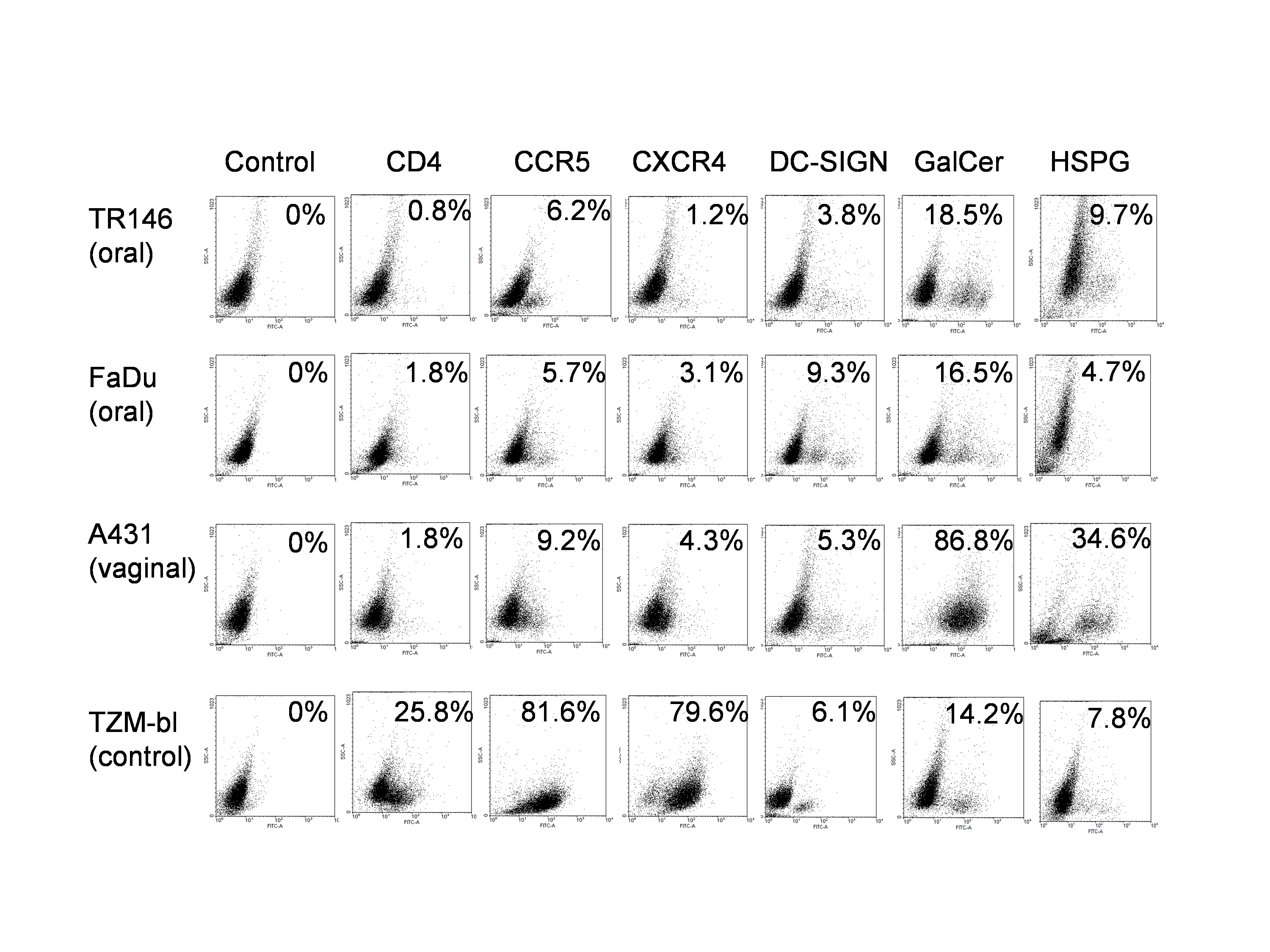

Supplement: Figure S1 — (TIF) [file pone.0098077.s001.tif]

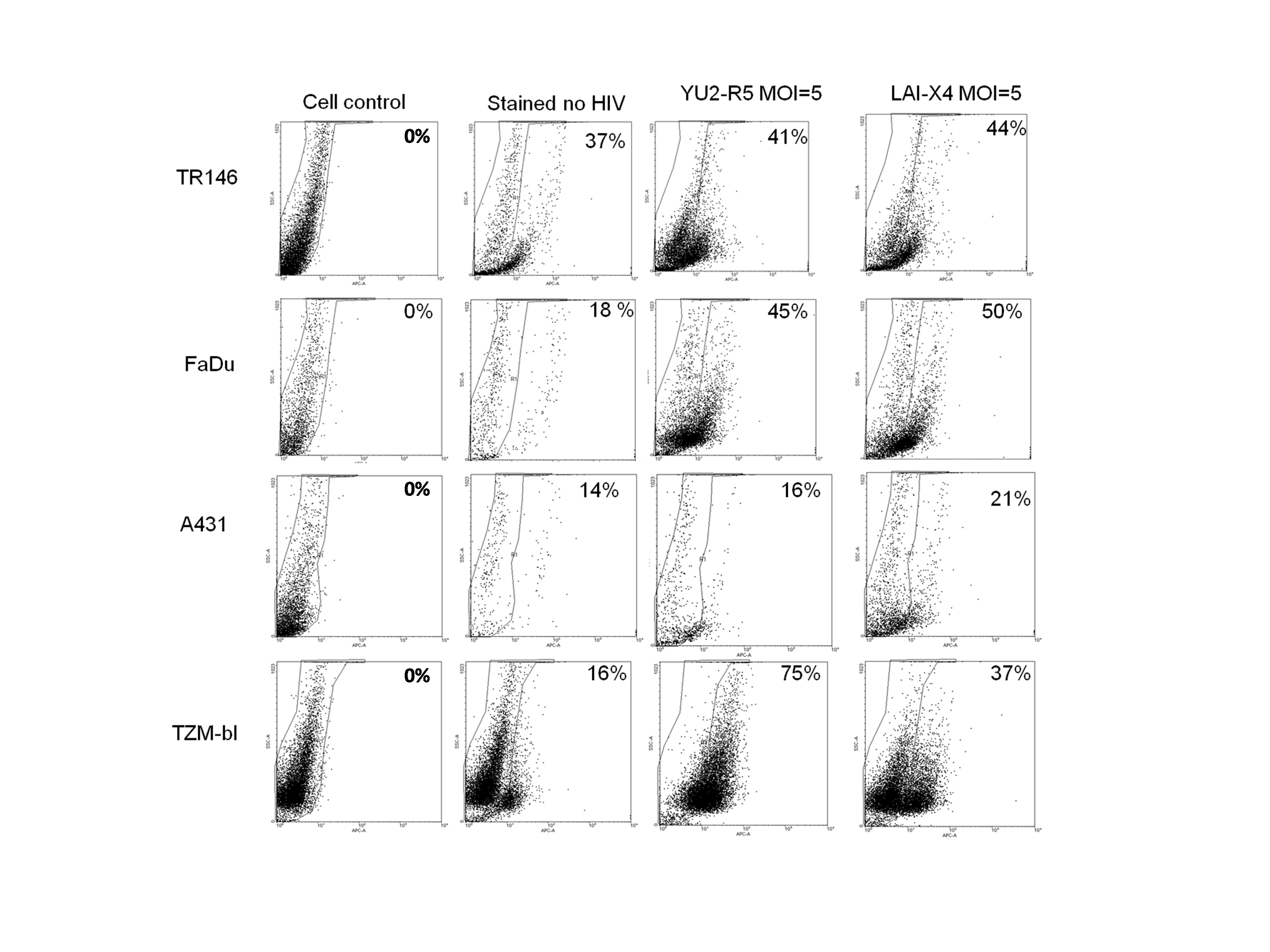

Supplement: Figure S2 — (TIF) [file pone.0098077.s002.tif]

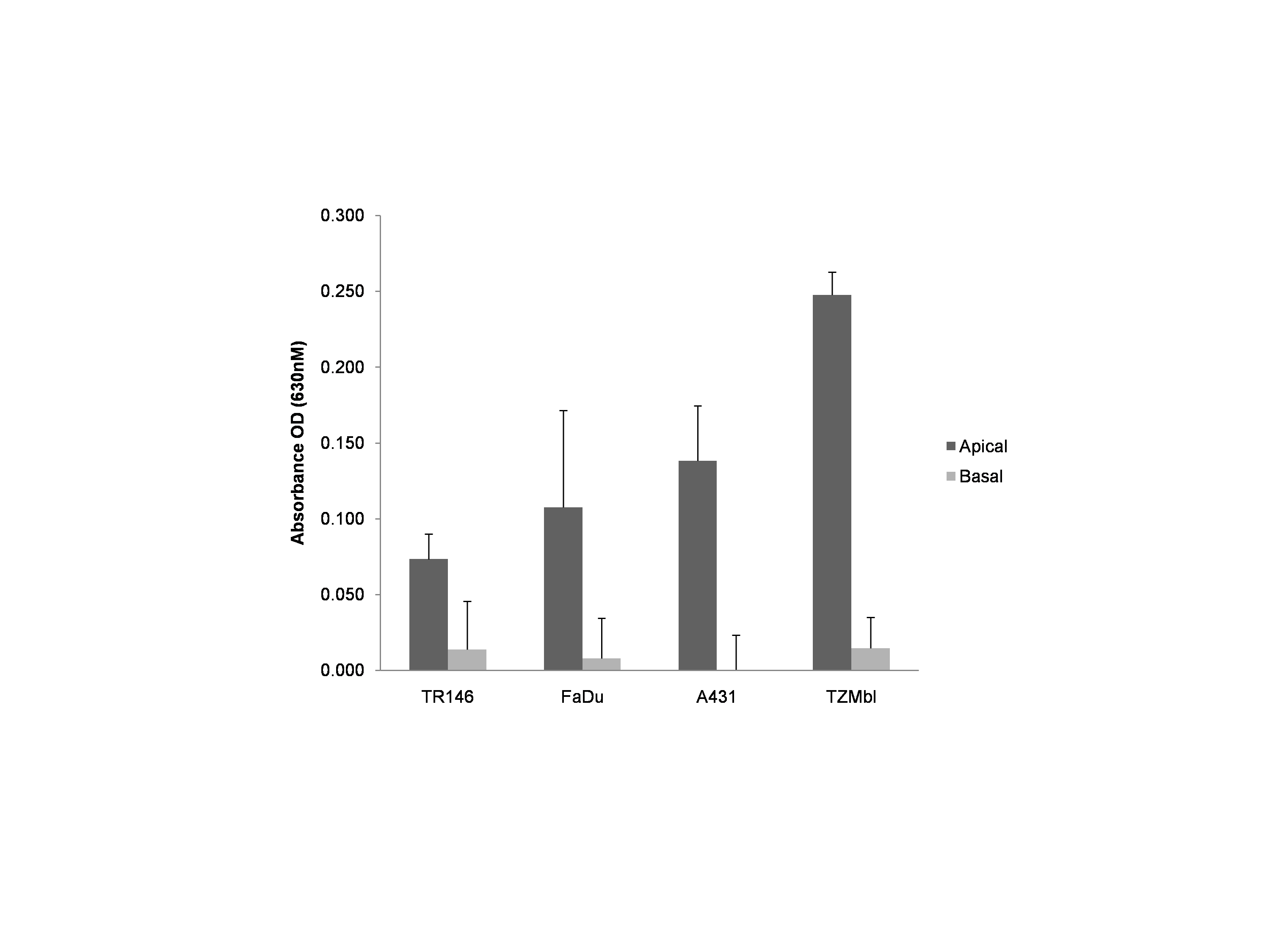

Supplement: Figure S3 — (TIF) [file pone.0098077.s003.tif]
