# Supplementary figures and images for: Correction: Oral and Vaginal Epithelial Cell Lines Bind and Transfer Cell-Free Infectious HIV-1 to Permissive Cells but Are Not Productively Infected
Source: PLoS One. 2020 Feb 19;15(2):e0229553. doi: 10.1371/journal.pone.0229553 (PMC7029853; doi:10.1371/journal.pone.0229553)

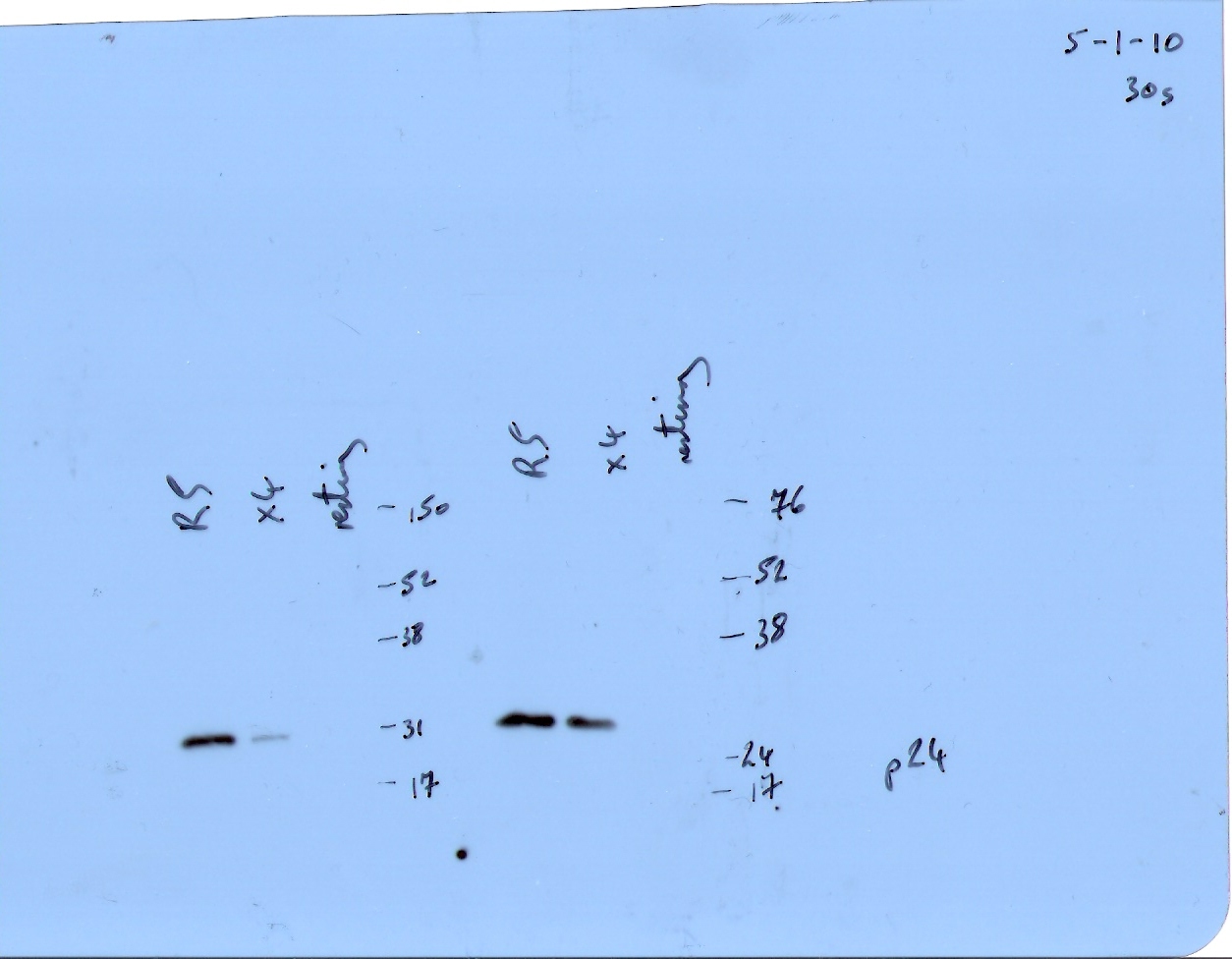

Supplement: S1 File — The image on the right, with aspect ratio adjusted to compress the image vertically, is shown in the published figure. (JPG) [file pone.0229553.s001.jpg]

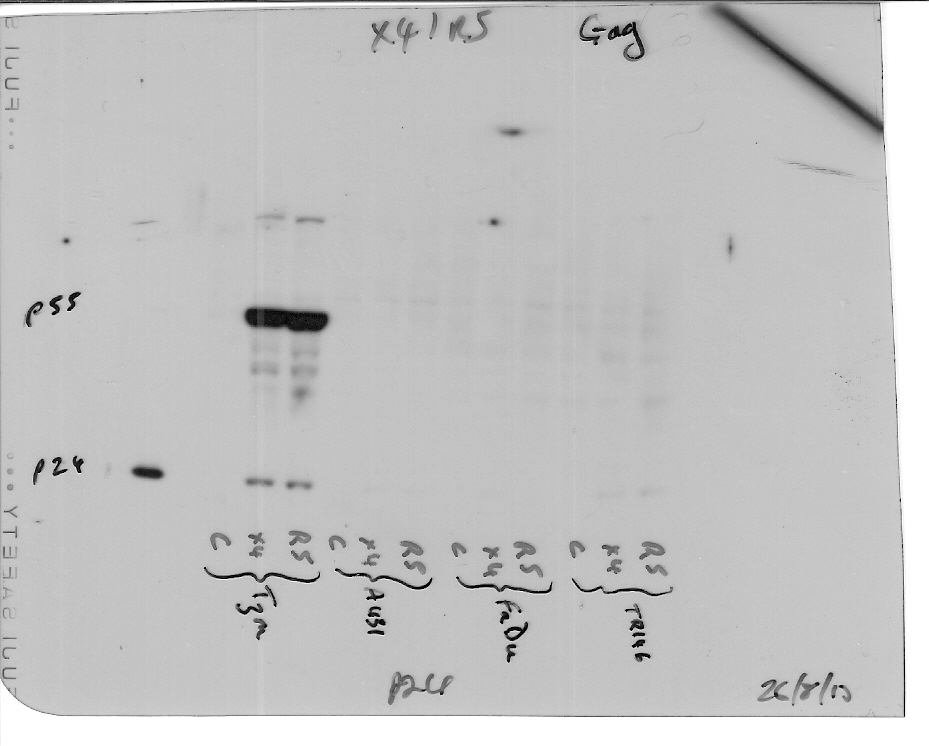

Supplement: S2 File — (JPG) [file pone.0229553.s002.jpg]

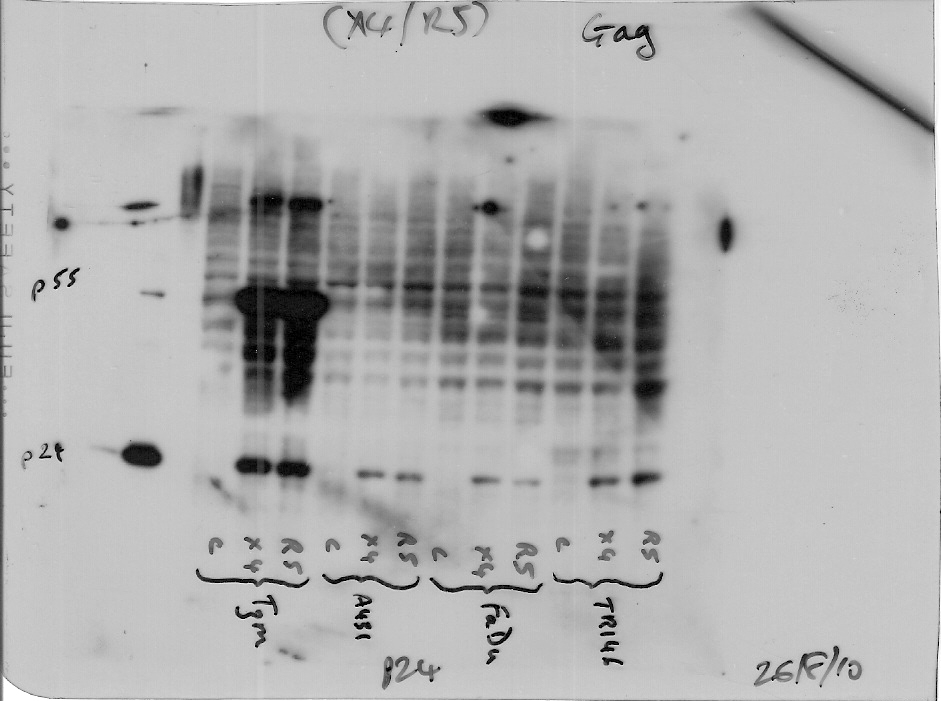

Supplement: S3 File — (JPG) [file pone.0229553.s003.jpg]

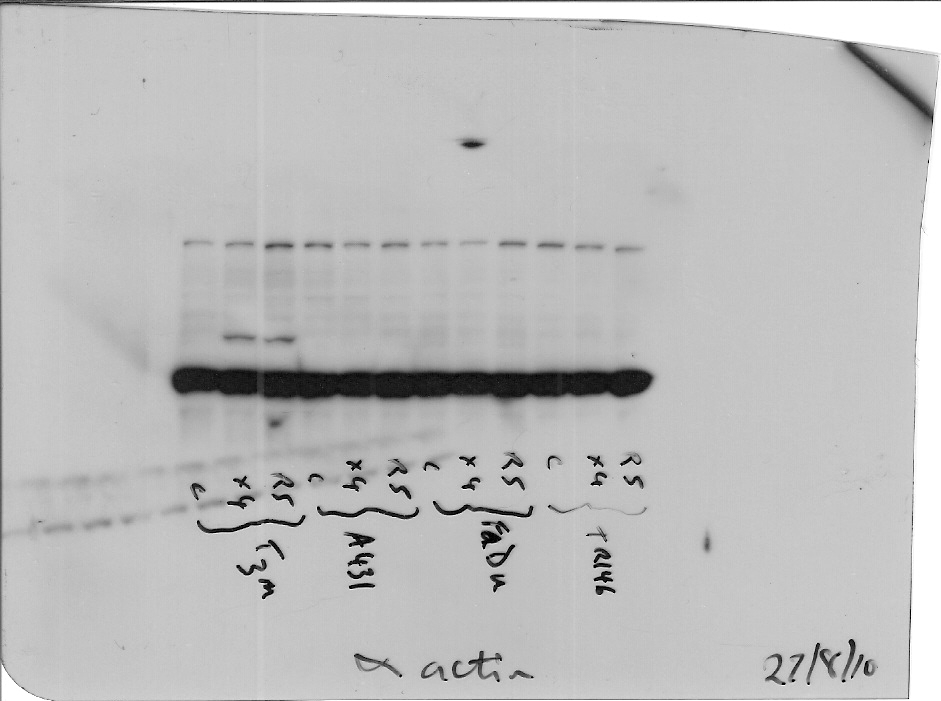

Supplement: S4 File — (JPG) [file pone.0229553.s004.jpg]

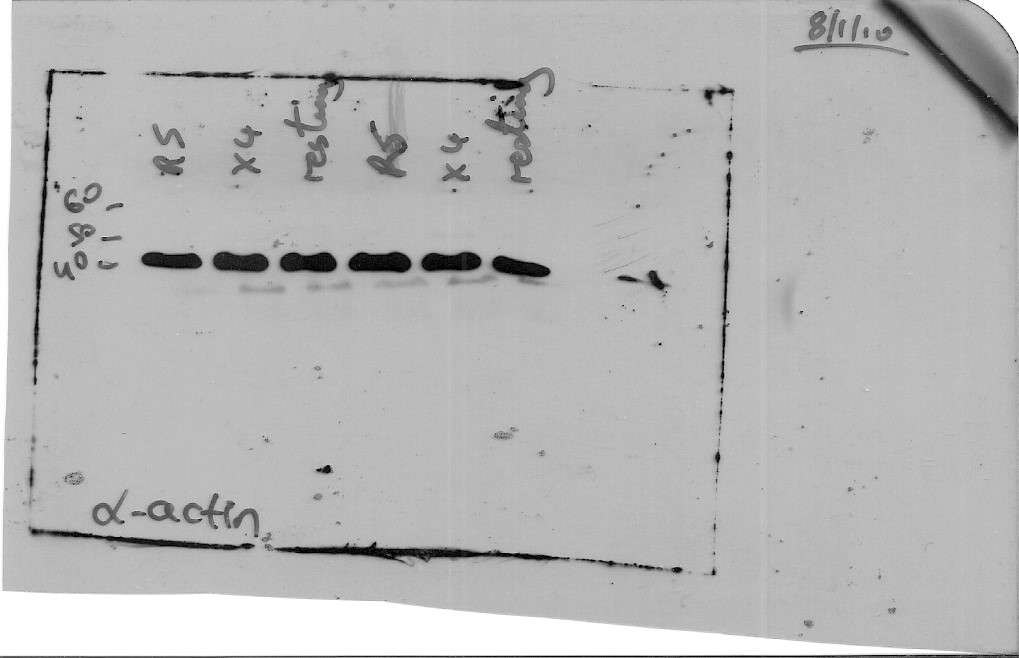

Supplement: S5 File — Data for the primary cell p24 and α-actin experiments were obtained using blots prepared with the same protein preparations. (JPG) [file pone.0229553.s005.jpg]
